# Author Correction: Herbivorous turtle ants obtain essential nutrients from a conserved nitrogen-recycling gut microbiome

**DOI:** 10.1038/s41467-018-04935-w

**Published:** 2018-06-19

**Authors:** Yi Hu, Jon G. Sanders, Piotr Łukasik, Catherine L. D’Amelio, John S. Millar, David R. Vann, Yemin Lan, Justin A. Newton, Mark Schotanus, Daniel J. C. Kronauer, Naomi E. Pierce, Corrie S. Moreau, John T. Wertz, Philipp Engel, Jacob A. Russell

**Affiliations:** 1 Department of Biology, Drexel University, Philadelphia, PA 19104 USA; 2 Department of Organismic and Evolutionary Biology, Harvard University, Cambridge, MA 02138 USA; 3 Department of Pediatrics, The University of California San Diego, La Jolla, CA 92093 USA; 4 Department of Medicine, Institute of Diabetes, Obesity and Metabolism, University of Pennsylvania, Philadelphia, PA 19104 USA; 5 Department of Earth and Environmental Science, University of Pennsylvania, Philadelphia, PA 19104 USA; 6 School of Biomedical Engineering, Science and Health systems, Drexel University, Philadelphia, PA 19104 USA; 7 Department of Biology, Calvin College, Grand Rapids, MI 49546 USA; 8 Laboratory of Social Evolution and Behavior, The Rockefeller University, New York, NY 10065 USA; 9 Department of Science and Education, Field Museum of Natural History, Chicago, IL 60605 USA; 10 Department of Fundamental Microbiology, University of Lausanne, 1015 Lausanne, Switzerland

Correction to: *Nature Communications*: 10.1038/s41467-018-03357-y, published online 06 Mar 2018

The originally published version of the Supplementary Information file associated with this Article contained an error in Supplementary Figure 3. Panel b was inadvertently replaced with a duplicate of panel a. The error has now been fixed and the corrected version of the Supplementary Information PDF is available to download from the HTML version of the Article.

