# Supplementary material for: Herbivorous turtle ants obtain essential nutrients from a conserved nitrogen-recycling gut microbiome
Source: Nat Commun. 2018 Mar 6;9:964. doi: 10.1038/s41467-018-03357-y (PMC5840417; doi:10.1038/s41467-018-03357-y)
Supplement: Supplementary file 1 — Supplementary Information [file 41467_2018_3357_MOESM1_ESM.pdf]

Description of Supplementary Files

File name: Supplementary Information
Description: Supplementary Figures, Supplementary Tables, Supplementary Methods and Supplementary Reference.

File name: Supplementary Data 1
Description: Genes from N-metabolic pathways in 18 *Cephalotes* ant gut microbiota and their distribution in different bacterial bins. B, R, O, P, X, C, F, S and H refer to Burkholderiales, Rhizobiales, Opitutales, Pseudomonadales, Xanthomonadales, Campylobacterales, Flavobacteriales, Sphingobacteriales and Hymenopera bins, respectively.

File name: Supplementary Data 2
Description: The distribution of genes from N-metabolic pathways in the 14 genomes of bacteria isolated from *C. varians* and *C. rohweri*.

File name: Supplementary Data 3
Description: Summary of scaffolds assigned to 11 bins in PL010 *C. varians* metagenome

File name: Supplementary Data 4
Description: The distribution of genes from N-metabolic pathways in the 11 bins generated based on the metagenome of *C. varians* colony PL010.

File name: Supplementary Data 5
Description: Results of *in vitro* urea production assays. Error bars indicate standard deviation.

File name: Supplementary Data 6
Description: Information of samples and fraction of the first isotopic peak abundance (M+1 abundance (fraction %)) of amino acids in the feeding experiments with ^{15}N-labelled urea and ^{13}C/^{15}N-labeled glutamate. The first isotopic peak represents the abundance of naturally occurring amino acids containing heavy isotopes.

File name: Supplementary Data 7
Description: OTU table from *C. varians* gut community samples used in feeding experiments with ^{15}N-labeled glutamate. The columns correspond to samples and rows correspond to OTUs. Numbers represent read abundance for each OTU within each library. Also indicated are taxonomic classification for each OTU.

Supplementary Information

Herbivorous turtle ants obtain essential nutrients from a highly conserved nitrogen-recycling gut microbiome

Yi Hu, Jon G. Sanders, Piotr Łukasik, Catherine L. D'Amelio, John S. Millar, David R. Vann, Yemin Lan, Justin A. Newton, Mark Schotanus, Daniel J. C. Kronauer, Naomi E. Pierce, Corrie S. Moreau, John T. Wertz, Philipp Engel, Jacob A. Russell

Table of Contents

Supplementary Figures 1
Supplementary Tables 21
Supplementary Methods 30
- Assessing N-fixation 30
- Feeding experiments with ^{15}N-labeled urea and $^{13}C/^{15}N$-labeled glutamate 30
- qPCR and amplicon 16S rRNA sequencing to estimate antibiotic efficacy 32
- Amino acid analysis from ant hemolymph by gas-chromatography-mass spectrometry (GC-MS) 33
- DNA preparation for *C. varians* metagenomics, non-*C. varians* ants for metagenomics and cultured bacteria 34-35
- Genome and metagenome sequencing, assembly and annotation 36
- Genome binning using Anvi'o in conjunction with the CONCOCT 39
- Visualization of taxonomic composition of metagenomes based on coverage and %GC ... 40
- Fluorescence in situ hybridization 40
- Stable isotope data 41
- Assays to measure urea production (via allantoin) and urea degradation (into ammonia) . 41

Supplementary References 44

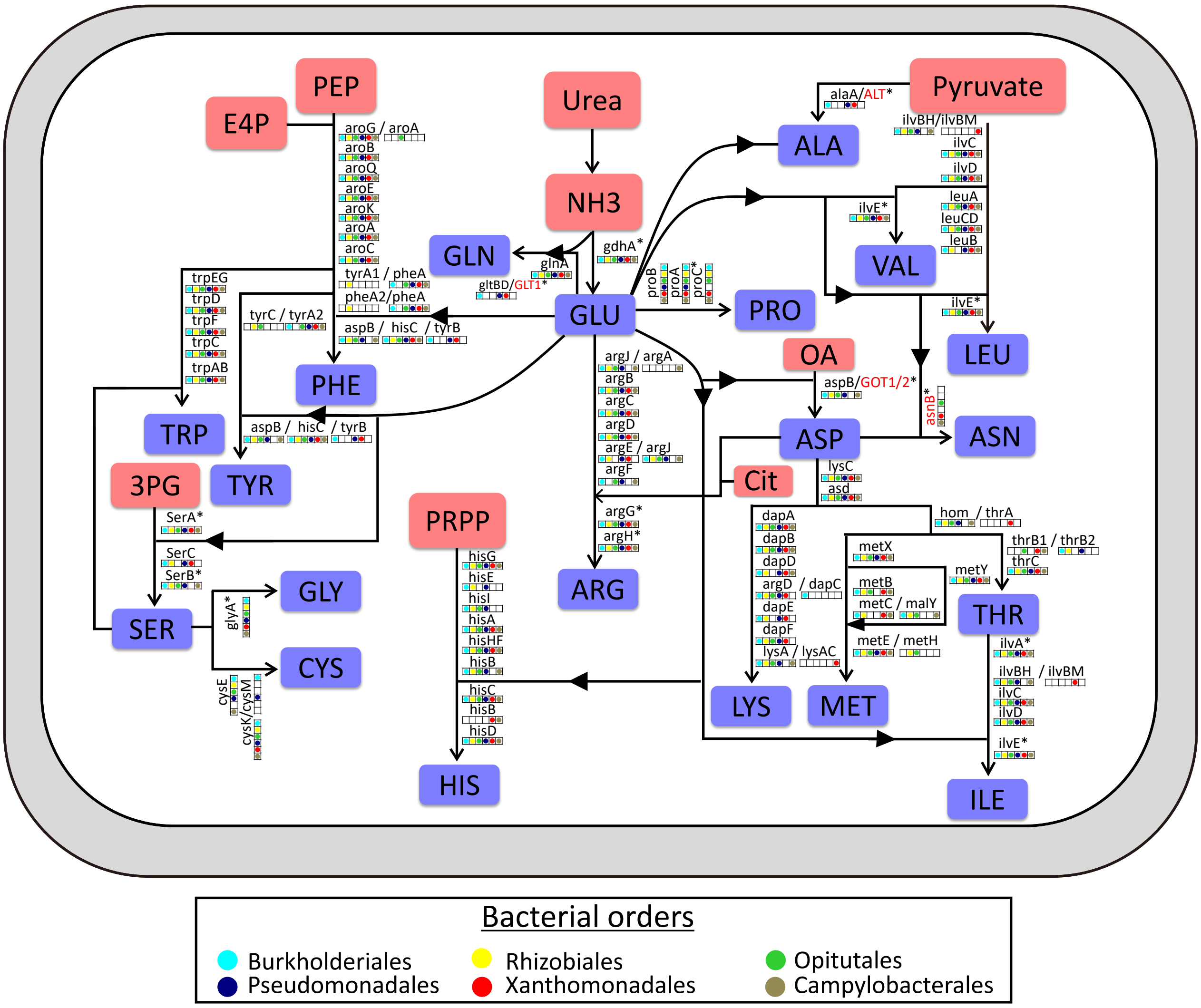

Supplementary Figure 1. Predicted essential amino acid biosynthetic pathways in the gut metagenome of *Cephalotes varians*. Names of genes not found in bacterial genomes are in red font. Asterisks indicated that genes were identified in the ant genome. Data are compiled from the metagenomes from colonies PL005 and PL010. 3PG, 3-phosphoglycerate; E4P, erythrose-4-phosphate; PEP, phosphoenolpyruvate; PRPP, phosphoribosyl pyrophosphate; OA, oxaloacetate; Cit, citrulline. Lines containing triangles near their midpoints allude to transamination reactions, where NH_3 groups from glutamate are donated to the given amino acid precursors. Arrows (i.e. with arrow heads at the end of a line) indicate carbon skeleton transformations of the given amino acid precursors.

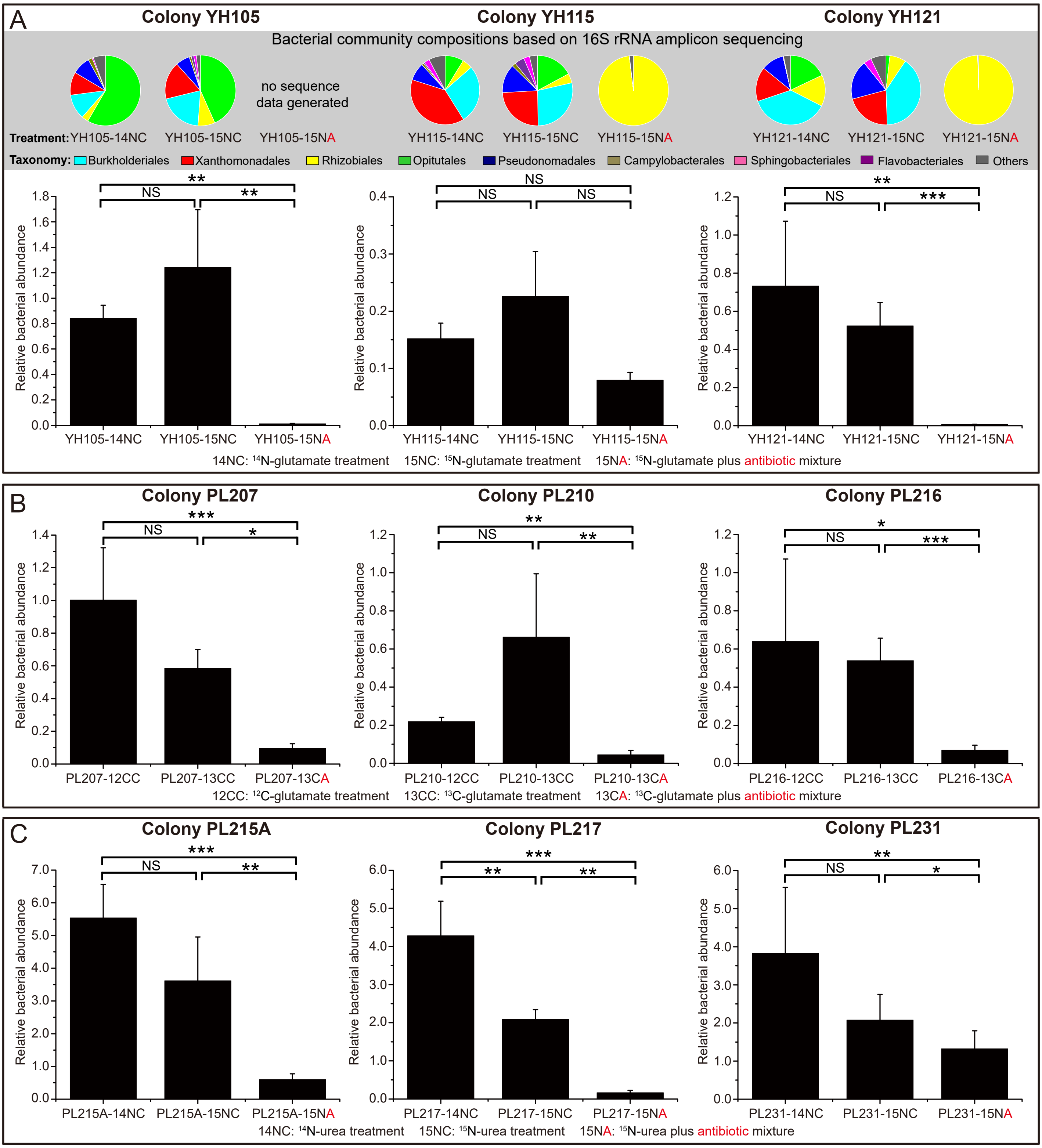

Supplementary Figure 2. Relative bacterial abundance in the ant groups under different dietary treatments in the ^{15}N labeled glutamate (A), ^{13}C labeled glutamate (B) and ^{15}N labeled urea feeding experiment (C). Relative bacterial abundance was determined by dividing bacterial 16S rRNA copy number estimates by one tenth of the total amount of bacterial 16S rRNA copy number estimates of the pooled gut DNA sample used for constructing the standard curve. 16S rRNA amplicon sequencing was performed only for ants in $^{15/14}N$ glutamate feeding experiment. Each pie chart shows average relative abundance for various symbionts sampled across replicate workers for each treatment. Information on the numbers of workers used for each treatment, and their parental colonies, is provided in **Supplementary Data 7**. Note that the approach used to obtain symbiont DNA for qPCR targeted a different set of tissues (i.e. gasters, which contain the crop, midgut, ileum, and rectum, along with non-gut, bacteria-free tissues) than those used to prepare DNA for shotgun metagenomics sequencing (i.e. dissected midgut and ileum tissues). Differing protocols, or perhaps taxon bias in the differing molecular methods, could account for differences seen in symbiont relative abundance in this figure and Fig. 3. Significant differences between different dietary treatments were determined by one-way ANOVA followed by Tukey's post-hoc test for normal data and Kruskal-Wallis tests followed by multiple pairwise comparisons using the Wilcoxon rank sum test for non-normal data (*P < 0.5, **P<0.01, ***P<0.001, NS = not statistically significant). Data are mean ± standard error.

A

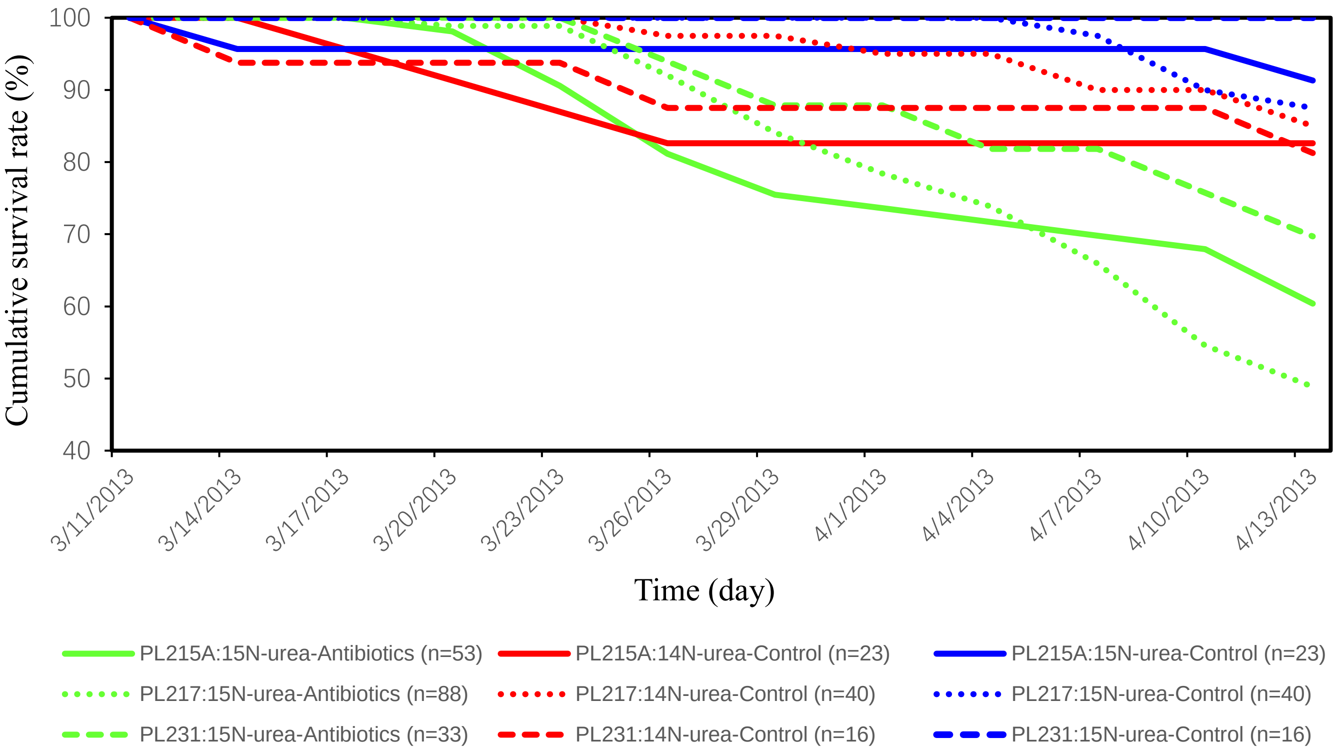

Cumulative survival rate (%)
100
90
80
70
60
50
40
3/11/2013
3/14/2013
3/17/2013
3/20/2013
3/23/2013
3/26/2013
3/29/2013
4/1/2013
4/4/2013
4/7/2013
4/10/2013
4/13/2013
Time (day)
PL215A:15N-urea-Antibiotics (n=53)
PL215A:14N-urea-Control (n=23)
PL215A:15N-urea-Control (n=23)
PL217:15N-urea-Antibiotics (n=88)
PL217:14N-urea-Control (n=40)
PL217:15N-urea-Control (n=40)
PL231:15N-urea-Antibiotics (n=33)
PL231:14N-urea-Control (n=16)
PL231:15N-urea-Control (n=16)

B

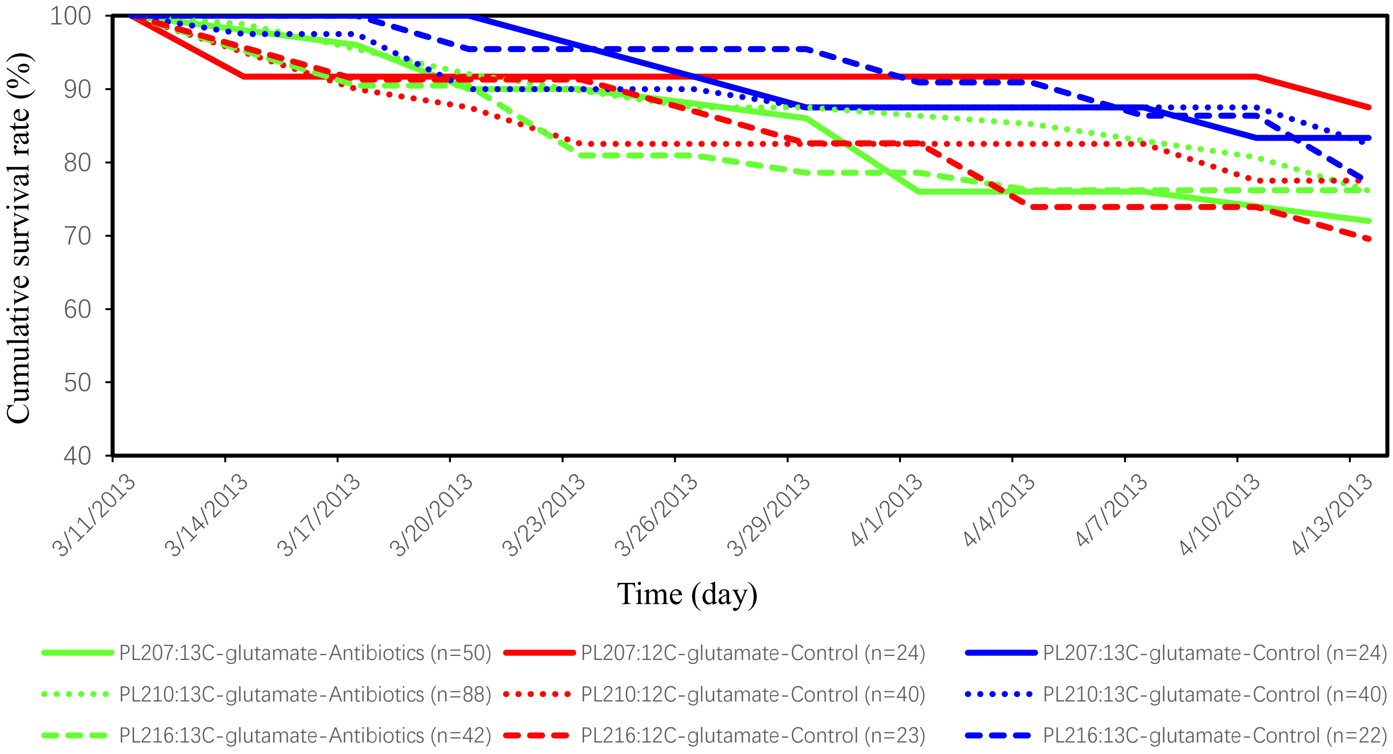

Cumulative survival rate (%)
100
90
80
70
60
50
40
3/11/2013
3/14/2013
3/17/2013
3/20/2013
3/23/2013
3/26/2013
3/29/2013
4/1/2013
4/4/2013
4/7/2013
4/10/2013
4/13/2013
Time (day)
PL207:13C-glutamate-Antibiotics (n=50)
PL207:12C-glutamate-Control (n=24)
PL207:13C-glutamate-Control (n=24)
PL210:13C-glutamate-Antibiotics (n=88)
PL210:12C-glutamate-Control (n=40)
PL210:13C-glutamate-Control (n=40)
PL216:13C-glutamate-Antibiotics (n=42)
PL216:12C-glutamate-Control (n=23)
PL216:13C-glutamate-Control (n=22)

Supplementary Figure 3. Survival of *Cephalotes varians* workers under different dietary treatments with isotope labeling of dietary urea (A) and dietary glutamate (B) with symbiont removal or maintenance. (A) Cox regression analysis for the workers fed on antibiotics (green lines) shows that disruption of gut microbiota significantly reduces survival (Wald statistic = 6.89, df = 1, P=0.0087 for colony PL215A; Wald statistic = 22.67, df = 1, P= 1.924e-06 for colony PL217; Wald statistic = 3.67, df = 1, P=0.0553 for colony PL231). (B) Cox regression analysis for the workers fed on antibiotics (green lines) shows that disruption of gut microbiota has no effect on survival of *C. varians* in the glutamate-feeding experiment (Wald statistic = 2.4, df = 1, P=0.1214 for colony PL207; Wald statistic = 0.29, df = 1, P= 0.5888 for colony PL210; Wald statistic = 0, df = 1, P=0.9882 for colony PL231).

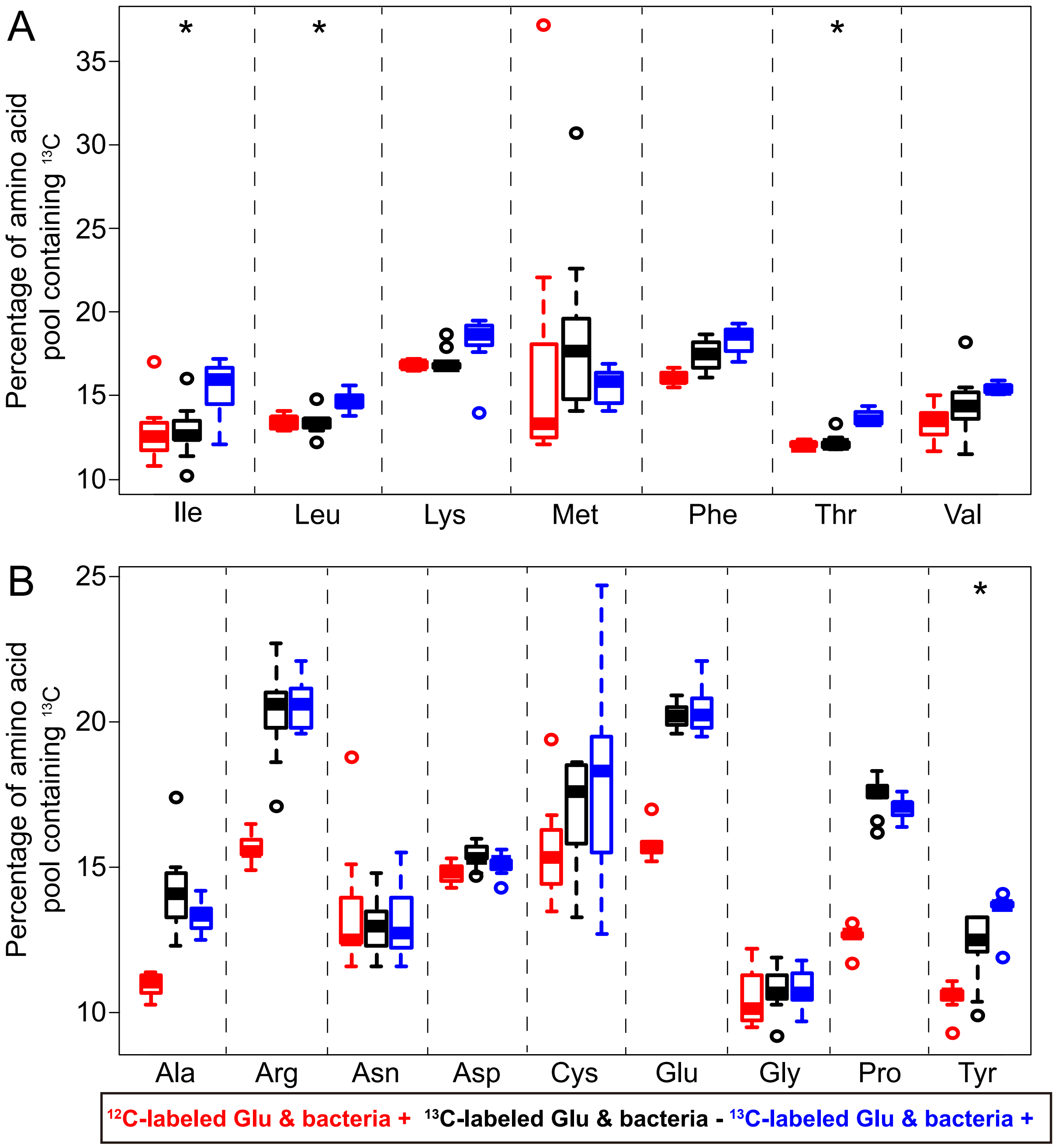

Supplementary Figure 4. Percentage of ^{13}C-labeling of free essential amino acids (A) and non-essential amino acids (B) in hemolymph of *Cephalotes varians* fed with ^{13}C-labeled glutamate. Asterisks indicated that ^{13}C in amino acids from ^{13}C-treated ants (blue) was significantly higher than in ants feeding on unlabeled glutamate (red) and in aposymbiotic ants feeding on ^{13}C-labeled glutamate (black) across three investigated colonies. Details on sample sizes for each treatment can be found in **Supplementary Data 6**. Note that each separate box plot graphs the median (center line), the interquartile range (i.e. from the top to the bottom of each box), values equal to 1.5 times the interquartile range (tops and bottoms of whiskers), along with outliers. Amino acids for which we observed significantly higher heavy isotope signal in the no antibiotic (blue) versus antibiotic (black) treatments, on the heavy isotope diet, are indicated with an asterisk. Statistics are described in the **Supplementary Methods** and **Supplementary Table 3**. In short, asterisks reveal results from Tukey's post-hoc tests (for normally distributed data after logit transformation) or Wilcoxon rank sum tests (for non-normal data after logit transformation).

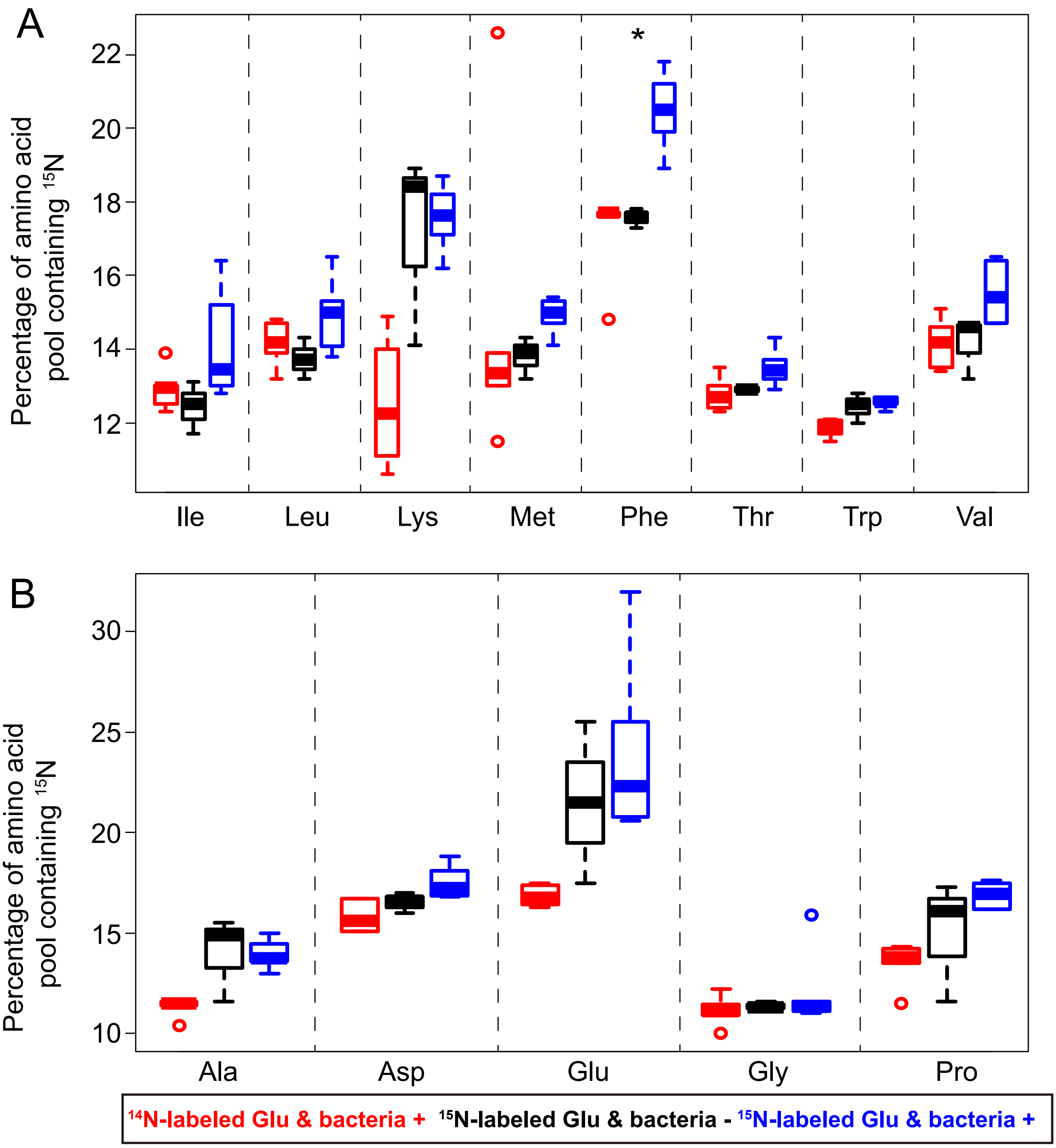

Supplementary Figure 5. Percentage of ^{15}N-labeling of free essential amino acids (A) and non-essential amino acids (B) in hemolymph of *Cephalotes varians* fed with ^{15}N-labeled glutamate. Asterisks indicated that ^{15}N in amino acids from ^{15}N-treated ants (blue) was significantly higher than in ants feeding on unlabeled glutamate (red) and in aposymbiotic ants feeding on ^{15}N-labeled glutamate (black) across three investigated colonies. Details on sample sizes for each treatment can be found in **Supplementary Data 6**. Note that each separate box plot graphs the median (center line), the interquartile range (i.e. from the top to the bottom of each box), values equal to 1.5 times the interquartile range (tops and bottoms of whiskers), along with outliers. Amino acids for which we observed significantly higher heavy isotope signal in the no antibiotic (blue) versus antibiotic (black) treatments, on the heavy isotope diet, are indicated with an asterisk. Statistics are described in the **Supplementary Methods** and **Supplementary Table 3**. In short, asterisks reveal results from Tukey's post-hoc tests (for normally distributed data after logit transformation) or Wilcoxon rank sum tests (for non-normal data after logit transformation).

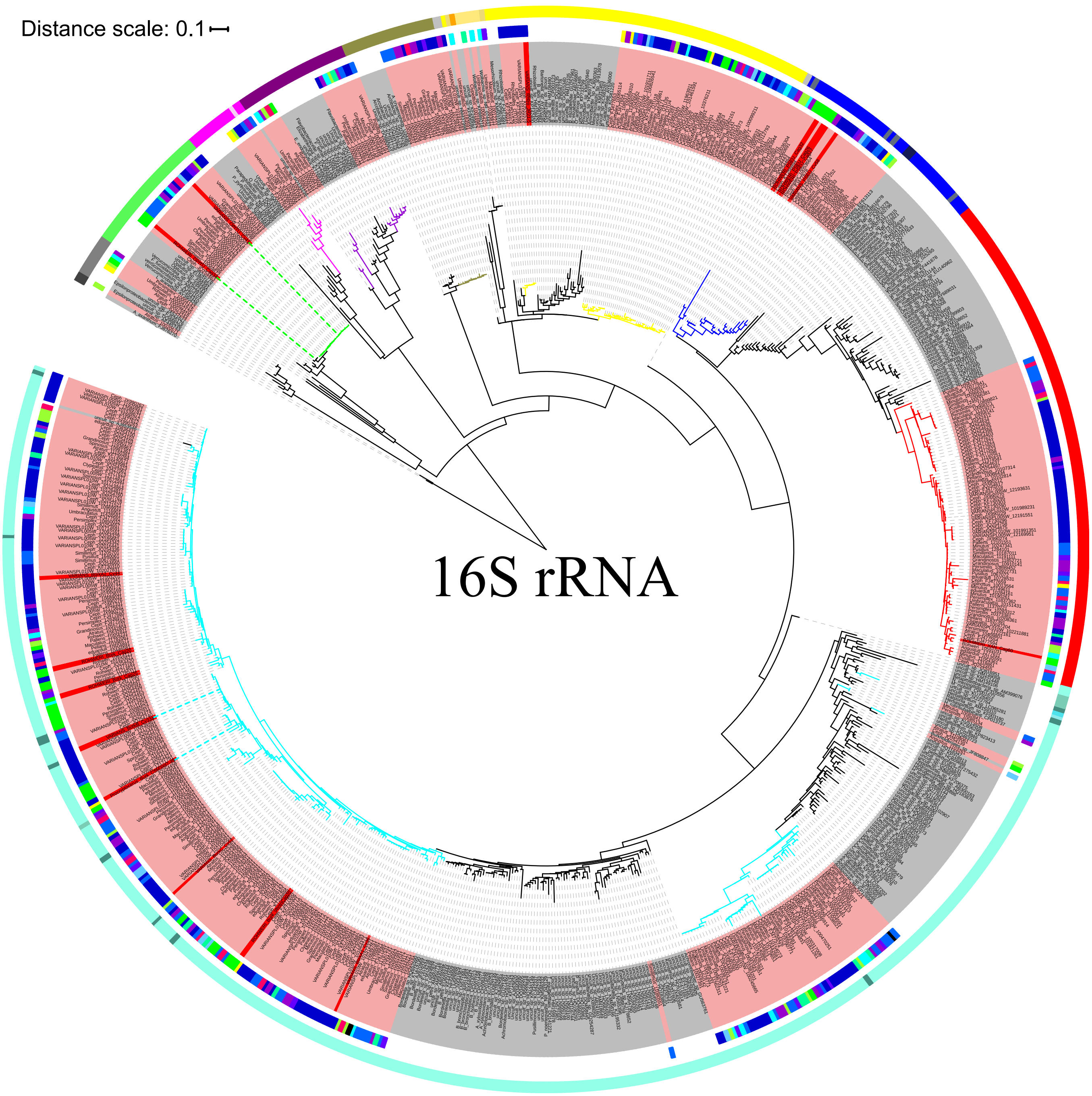

Taxonomy (outer circle)

Proteobacteria: Betaproteobacteria
- Burkholderiales
- Neisseriales
- Rhodocyclales
- Methylophiales
- unclassified Betaproteobacteria

Proteobacteria: Gammaproteobacteria
- Xanthomonadales
- Pseudomonadales
- Oceanospirillales
- unclassified Gammaproteobacteria

Proteobacteria: Alphaproteobacteria
- Rhizobiales
- Rhodospirillales
- Rickettsiales
- unclassified Alphaproteobacteria

Proteobacteria: Epsilonproteobacteria
- Campylobacterales

Proteobacteria
- unclassified Proteobacteria

Verrucomicrobia
- Opitutales

Bacteroidetes: Flavobacteria
- Flavobacteriales

Bacteroidetes: Sphingobacteria
- Sphingobacteriales

Bacteroidetes
- unclassified Bacteroidetes

Others
- unclassified Bacteria
- outgroup

Cephalotine ant species (middle circle)

C. atratus, *C. rowheri*, *C. clypeatus*, *C. simillimus*, *C. minustus*, *C. spinosus*, *C. pusillus*, *C. pellans*, *C. pallens*, *C. varians*, *C. angustus*, *C. umbraculatus*, *C. maculatus*, *C. grandinosus*, *C. persimilis*, *C. persimplex*, *C. eduarduli*, *Procryptocerus* sp.

Taxon name shading (inner circle)

- sequences from cultured isolates in this study
- metagenome derived sequences in this study
- non-Cephalotine bacteria

Supplementary Figure 6. Phylogeny of symbiont 16S rRNA genes reveals strong taxonomic conservation among worker-associated gut bacteria. Phylogenies of 16S rRNA nucleic acid sequences based on sequences extracted from 18 *Cephalotes* metagenomes and top BLAST hits.Rooted maximum likelihood phylogeny reveals nearly all *Cephalotes*-associates come from *Cephalotes*-specific clades. N-recycling bacteria identified through *in vitro* assays are emphasized with cyan or green lines connecting their branches to their strain names. Outer circle and branch colors: bacterial taxonomy. Middle circle colors: *Cephalotes* species groups. Inner circle: all red shading of taxon names reveals sequences coming from our metagenomic datasets, bright red shading of taxon names reveals cultured isolates and gray shading of taxon names represents bacteria found in hosts/habitats outside of Cephalotine ants.

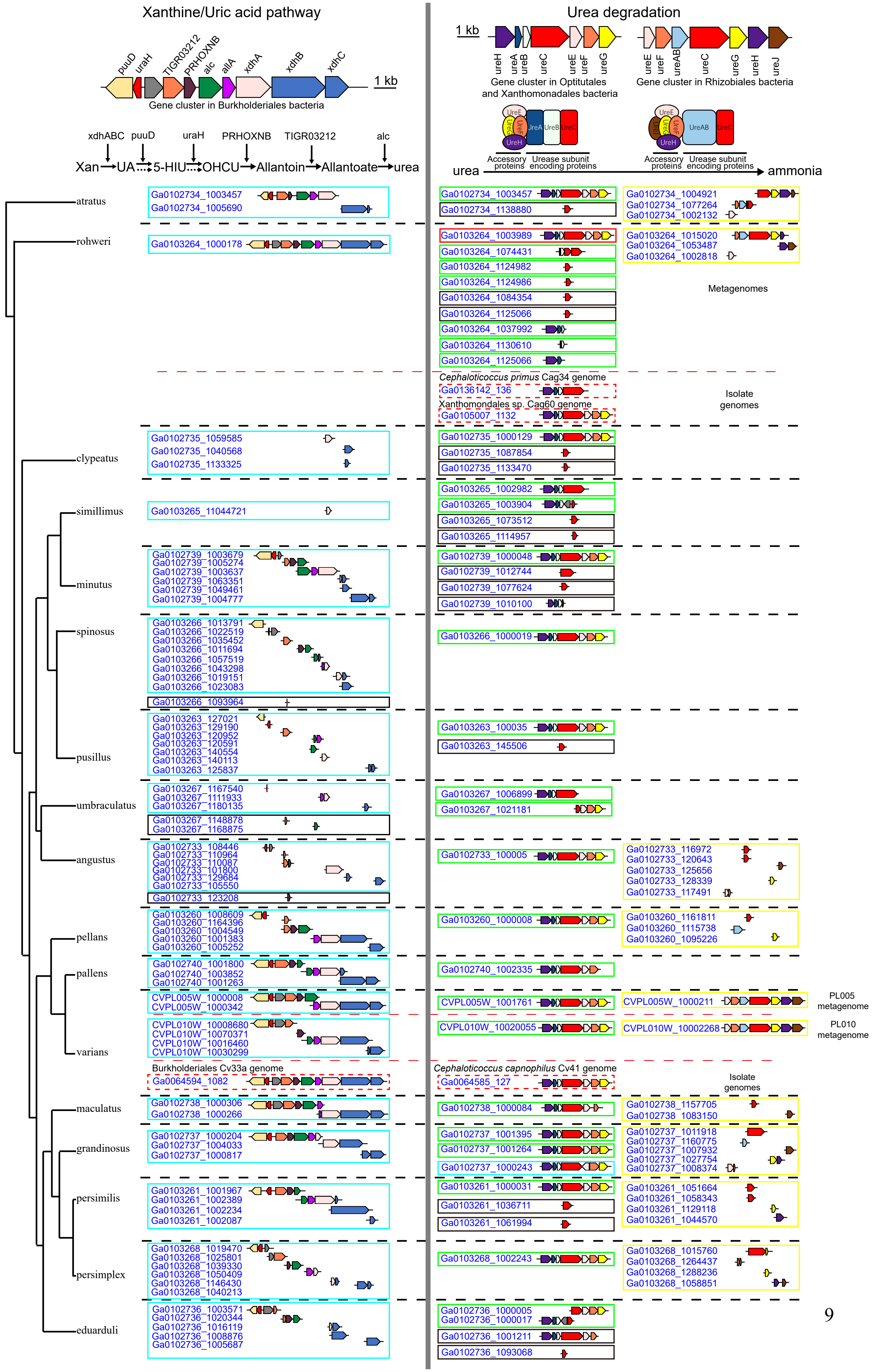

Xanthine/Uric acid pathway
puuD
uraH
TIGR03212
PRHOXNB
alc
allA
xdhA
xdhB
xdhC
1 kb
Gene cluster in Burkholderiales bacteria
xdhABC
puuD
uraH
PRHOXNB
TIGR03212
alc
Xan
UA
5-HIU
OHCU
Allantoin
Allantoate
urea
Urea degradation
1 kb
ureH
ureA
ureB
ureC
ureE
ureF
ureG
Gene cluster in Optitutales and Xanthomonadales bacteria
ureE
ureF
ureAB
ureC
ureG
ureH
ureJ
Gene cluster in Rhizobiales bacteria
UreE
UreG
UreF
UreH
UreA
UreB
UreC
UreAB
UreJ
Accessory proteins
Urease subunit encoding proteins
urea
ammonia
atratus
rohweri
clypeatus
simillimus
minutus
spinosus
pusillus
umbraculatus
angustus
pellans
pallens
varians
maculatus
grandinosus
persimilis
persimplex
eduarduli
Ga0102734_1003457
Ga0102734_1005690
Ga0102734_1003457
Ga0102734_1138880
Ga0102734_1004921
Ga0102734_1077264
Ga0102734_1002132
Ga0103264_1000178
Ga0103264_1003989
Ga0103264_1074431
Ga0103264_1124982
Ga0103264_1124986
Ga0103264_1084354
Ga0103264_1125066
Ga0103264_1037992
Ga0103264_1130610
Ga0103264_1125066
Ga0103264_1015020
Ga0103264_1053487
Ga0103264_1002818
Metagenomes
Cephaloticoccus primus Cag34 genome
Ga0136142_136
Xanthomondales sp. Cag60 genome
Ga0105007_1132
Isolate genomes
Ga0102735_1059585
Ga0102735_1040568
Ga0102735_1133325
Ga0102735_1000129
Ga0102735_1087854
Ga0102735_1133470
Ga0103265_11044721
Ga0103265_1002982
Ga0103265_1003904
Ga0103265_1073512
Ga0103265_1114957
Ga0102739_1003679
Ga0102739_1005274
Ga0102739_1003637
Ga0102739_1063351
Ga0102739_1049461
Ga0102739_1004777
Ga0102739_1000048
Ga0102739_1012744
Ga0102739_1077624
Ga0102739_1010100
Ga0103266_1013791
Ga0103266_1022519
Ga0103266_1035452
Ga0103266_1011694
Ga0103266_1057519
Ga0103266_1043298
Ga0103266_1019151
Ga0103266_1023083
Ga0103266_1093964
Ga0103266_1000019
Ga0103263_127021
Ga0103263_129190
Ga0103263_120952
Ga0103263_120591
Ga0103263_140554
Ga0103263_140113
Ga0103263_125837
Ga0103263_100035
Ga0103263_145506
Ga0103267_1167540
Ga0103267_1111933
Ga0103267_1180135
Ga0103267_1148878
Ga0103267_1168875
Ga0103267_1006899
Ga0103267_1021181
Ga0102733_108446
Ga0102733_110964
Ga0102733_110087
Ga0102733_101800
Ga0102733_129684
Ga0102733_105550
Ga0102733_123208
Ga0102733_100005
Ga0102733_116972
Ga0102733_120643
Ga0102733_125656
Ga0102733_128339
Ga0102733_117491
Ga0103260_1008609
Ga0103260_1164396
Ga0103260_1004549
Ga0103260_1001383
Ga0103260_1005252
Ga0103260_1000008
Ga0103260_1161811
Ga0103260_1115738
Ga0103260_1095226
Ga0102740_1001800
Ga0102740_1003852
Ga0102740_1001263
Ga0102740_1002335
CVPL005W_1000008
CVPL005W_1000342
CVPL005W_1001761
CVPL005W_1000211
PL005 metagenome
CVPL010W_10008680
CVPL010W_10070371
CVPL010W_10016460
CVPL010W_10030299
CVPL010W_10020055
CVPL010W_10002268
PL010 metagenome
Burkholderiales Cv33a genome
Ga0064594_1082
Cephaloticoccus capnophilus Cv41 genome
Ga0064585_127
Isolate genomes
Ga0102738_1000306
Ga0102738_1000266
Ga0102738_1000084
Ga0102738_1157705
Ga0102738_1083150
Ga0102737_1000204
Ga0102737_1004033
Ga0102737_1000817
Ga0102737_1001395
Ga0102737_1001264
Ga0102737_1000243
Ga0102737_1011918
Ga0102737_1160775
Ga0102737_1007932
Ga0102737_1027754
Ga0102737_1008374
Ga0103261_1001967
Ga0103261_1002389
Ga0103261_1002234
Ga0103261_1002087
Ga0103261_1000031
Ga0103261_1036711
Ga0103261_1061994
Ga0103261_1051664
Ga0103261_1058343
Ga0103261_1129118
Ga0103261_1044570
Ga0103268_1019470
Ga0103268_1025801
Ga0103268_1039330
Ga0103268_1050409
Ga0103268_1146430
Ga0103268_1040213
Ga0103268_1002243
Ga0103268_1015760
Ga0103268_1264437
Ga0103268_1288236
Ga0103268_1058851
Ga0102736_1003571
Ga0102736_1020344
Ga0102736_1016119
Ga0102736_1008876
Ga0102736_1005687
Ga0102736_1000005
Ga0102736_1000017
Ga0102736_1001211
Ga0102736_1093068

Supplementary Figure 7. Conserved clusters of genes involved in uric acid degradation and urea degradation pathways across 17 *Cephalotes* ant species. A cladogram based on reported relationships[12] is shown on the left. Names and functions of genes in the uric acid degradation and urea gene operons are given at the top of the figure. The arrow with dashed lines represents ant host derived metabolic steps. The gene structure of each operon was shown in all 18 metagenomes, with the left panel indicating Xanthine/Uric acid degradation gene operons and the right panel indicating Urea degradation gene operons. Each gene operon was labelled by the corresponding scaffold ID and was highlighted by a box colored by the bacterial orders to which they were binned. For some hosts (*C. varians* and *C. rohweri*) we present data from cultured isolate genome sequencing; such findings are indicated with labeling at right, while distinctions between the two metagenomes from *C. varians* are indicated at the right as well.

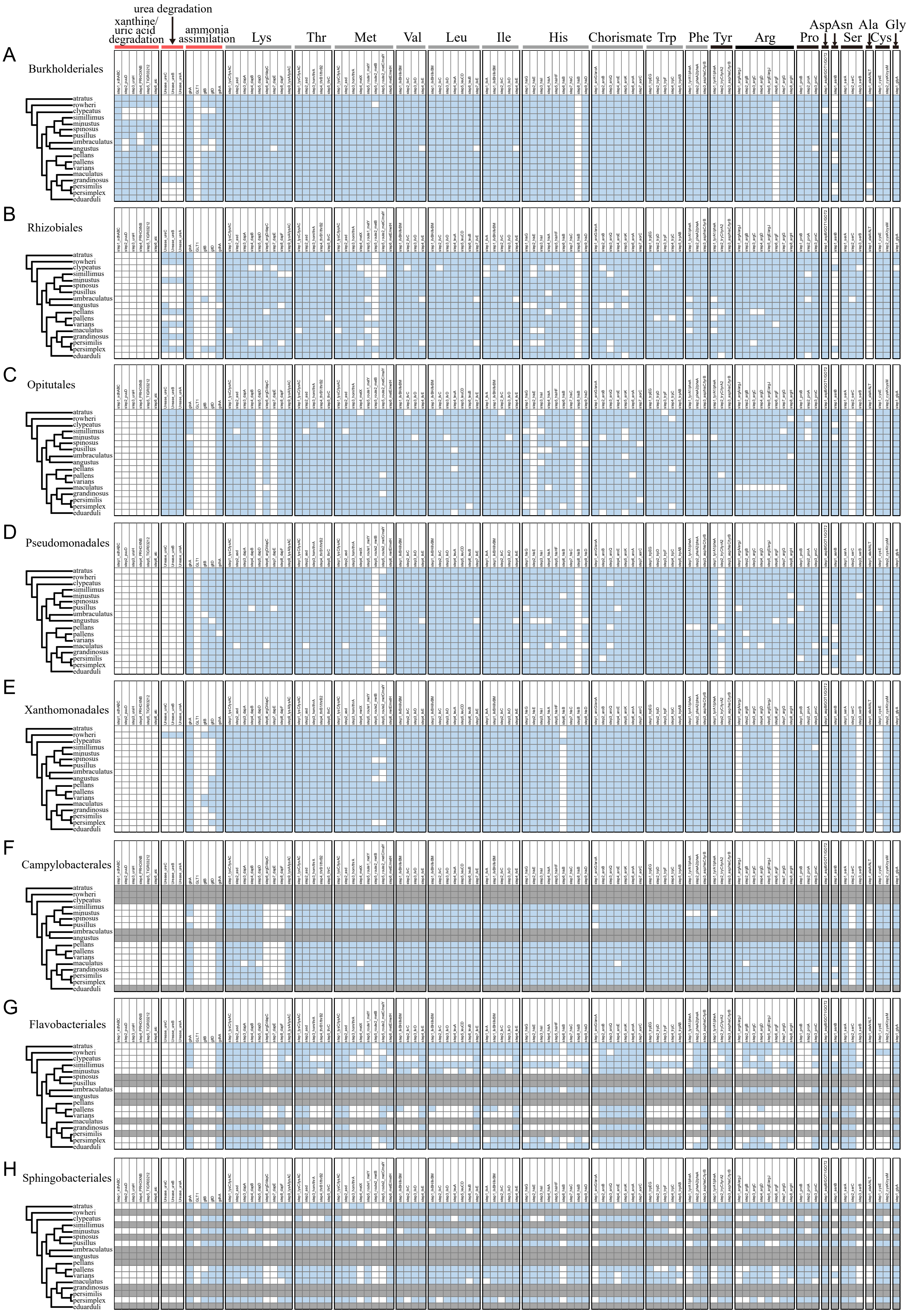
urea degradation
xanthine/ uric acid degradation
ammonia assimilation
Lys
Thr
Met
Val
Leu
Ile
His
Chorismate
Trp
Phe
Tyr
Arg
Pro
Asp
Asn
Ser
Ala
Cys
Gly
A
Burkholderiales
B
Rhizobiales
C
Opitutales
D
Pseudomonadales
E
Xanthomonadales
F
Campylobacterales
G
Flavobacteriales
H
Sphingobacteriales
atratus
rowheri
clypeatus
simillimus
minustus
spinosus
pusillus
umbraculatus
angustus
pellans
pallens
varians
maculatus
grandinosus
persimilis
persimplex
eduarduli

Supplementary Figure 8. Presence or absence of genes involved in pathways of xanthine/uric acid degradation, urea degradation, ammonia assimilation and amino acid synthesis for eight bacterial bins in each of the gut metagenome of 18 *Cephalotes.* Symbionts hail from the orders Burkholderiales (A), Rhizobiales (B), Opitutales (C), Pseudomonadales (D), Xanthomonadales (E), Campylobacterales (F), Flavobacteriales (G) and Sphingobacteriales (H). White and blue in each heatmap respectively represent the absence and presence of genes associated with the focal metabolic pathways. If total length of scaffolds belonging to a specific bacterial taxon from one metagenomic dataset is less than 50% of the total length of the same taxa draft genome assembled from metagenome, gray bars denote the lack of pathway information for the core bacterial bins of *Cephalotes* ants. A cladogram based on previously published relationships of 18 *Cephalotes* ants[12] is shown to the left of each panel. Common ancestry traces back to roughly 46 million years.

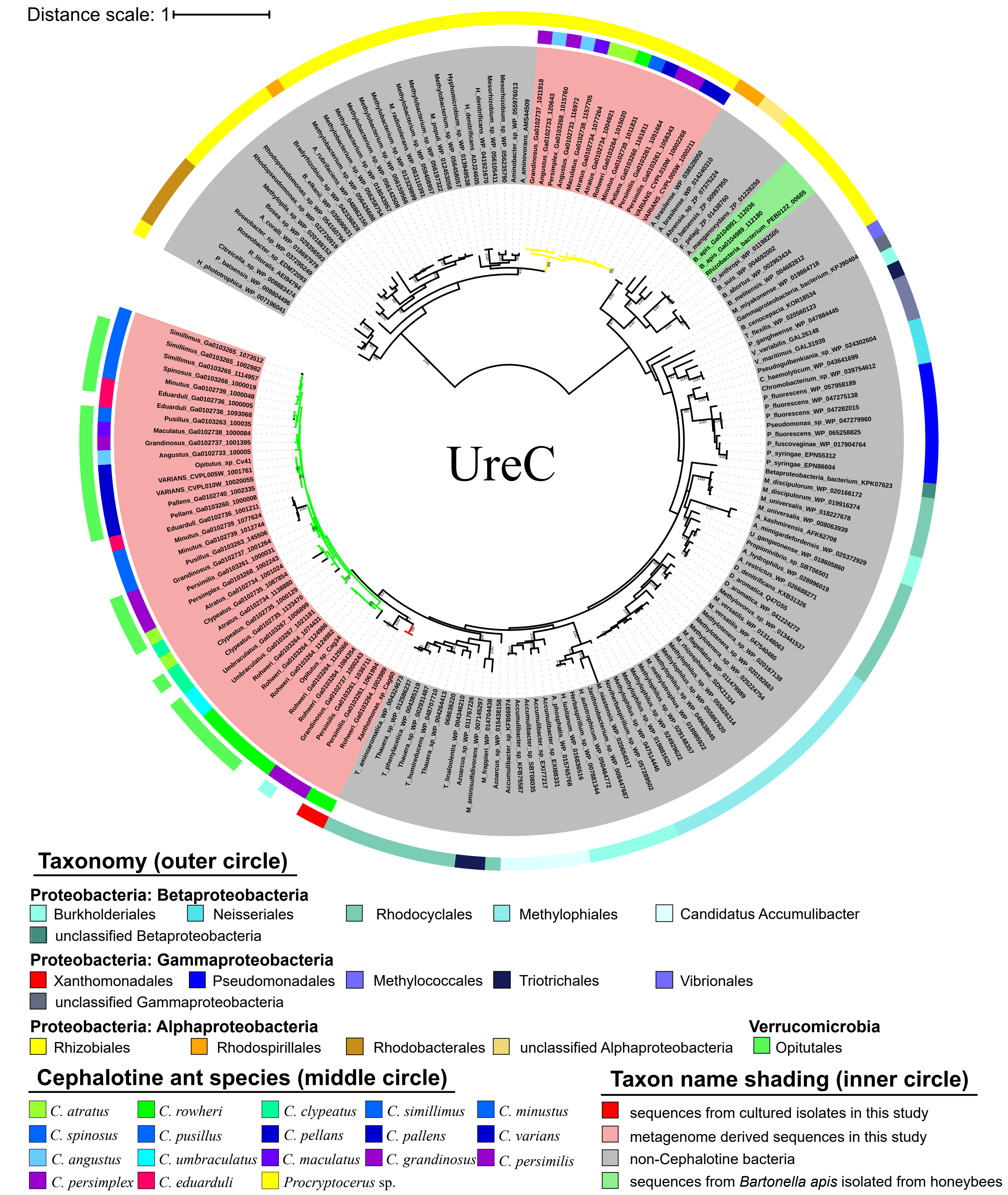

Supplementary Figure 9. Phylogenety of symbiont UreC proteins reveals patterns of convergent functional evolution among worker-associated gut bacteria. Phylogenies of UreC proteins based on sequences extracted from 18 *Cephalotes* metagenomes and top BLAST hits. Rooted maximum likelihood phylogeny reveals nearly all *Cephalotes*-associates come from *Cephalotes*-specific clades. Outer circle and branch colors: bacterial taxonomy. A lack of shading in the outer circle, for *Cephalotes*-derived sequences, revealed that ureC genes fell on contigs that could not be assigned to bacterial phyla or any lower taxa. Middle circle colors: *Cephalotes* species groups. Inner circle: all red shading of taxon names reveals sequences coming from our metagenomic datasets, bright red shading of taxon names reveals cultured isolates, gray shading of taxon names represents bacteria found in hosts/habitats outside of Cephalotine ants and green shadings of taxon names reveals sequences from *Bartonella apis* isolated from honeybees.

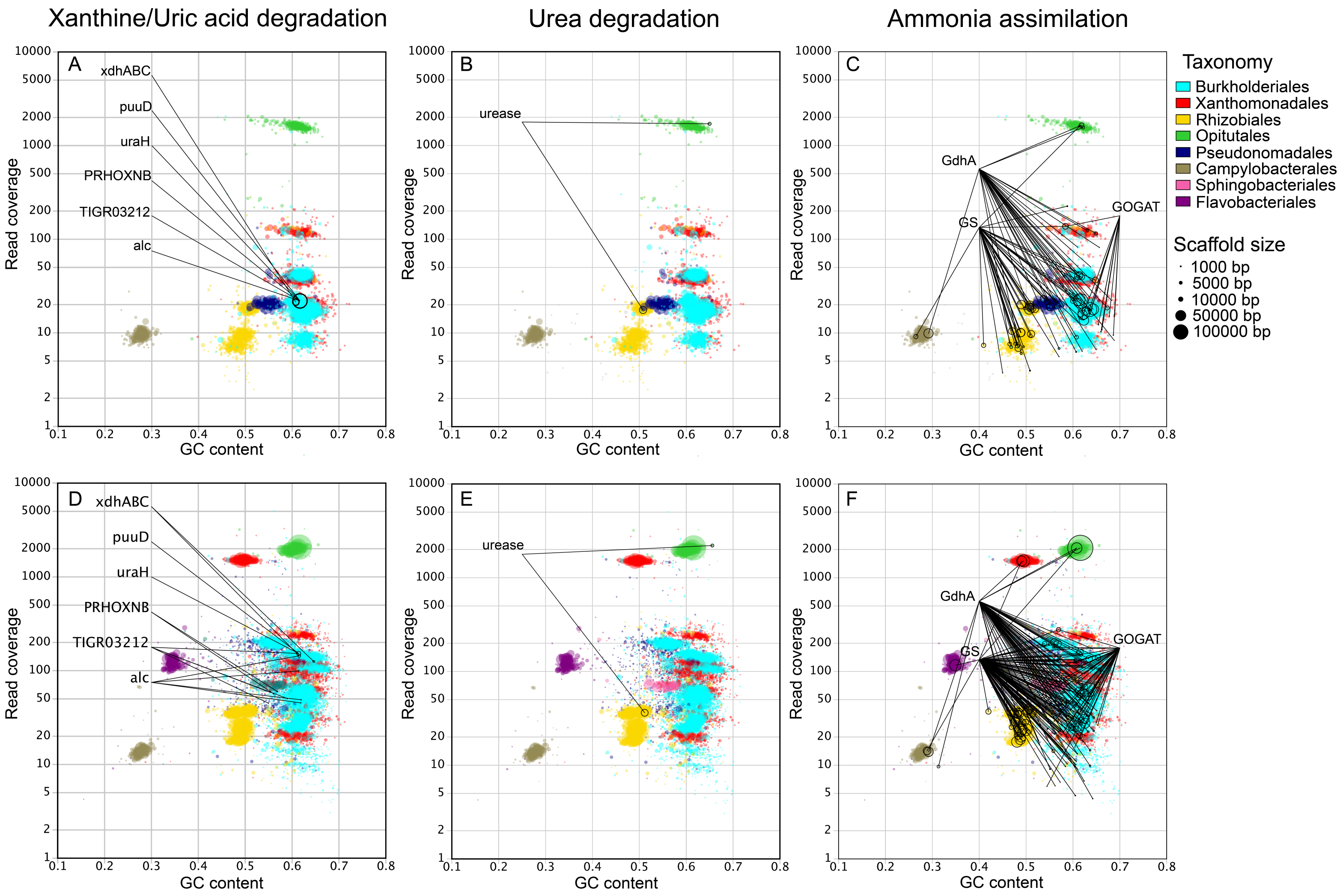

Supplementary Figure 10. Distribution of scaffolds containing genes in the N-metabolic pathways in taxon-annotated GC-coverage (TAGC) plots for the metagenomes of *Cephalotes varians*. Individual scaffolds are plotted based on their GC content (x-axis) and their read coverage (y-axis, logarithmic scale). Scaffolds are colored based on the taxonomic order they were assigned to as described in the text. (A) and (D) Scaffolds containing genes in uric acid degradation pathways were highlighted in the TAGC plots of colony PL005 (top) and PL010 (bottom). (B) and (E) Scaffolds containing genes in urea degradation pathways were similarly highlighted, as were those containing genes involved in ammonia assimilation (C) and (F).

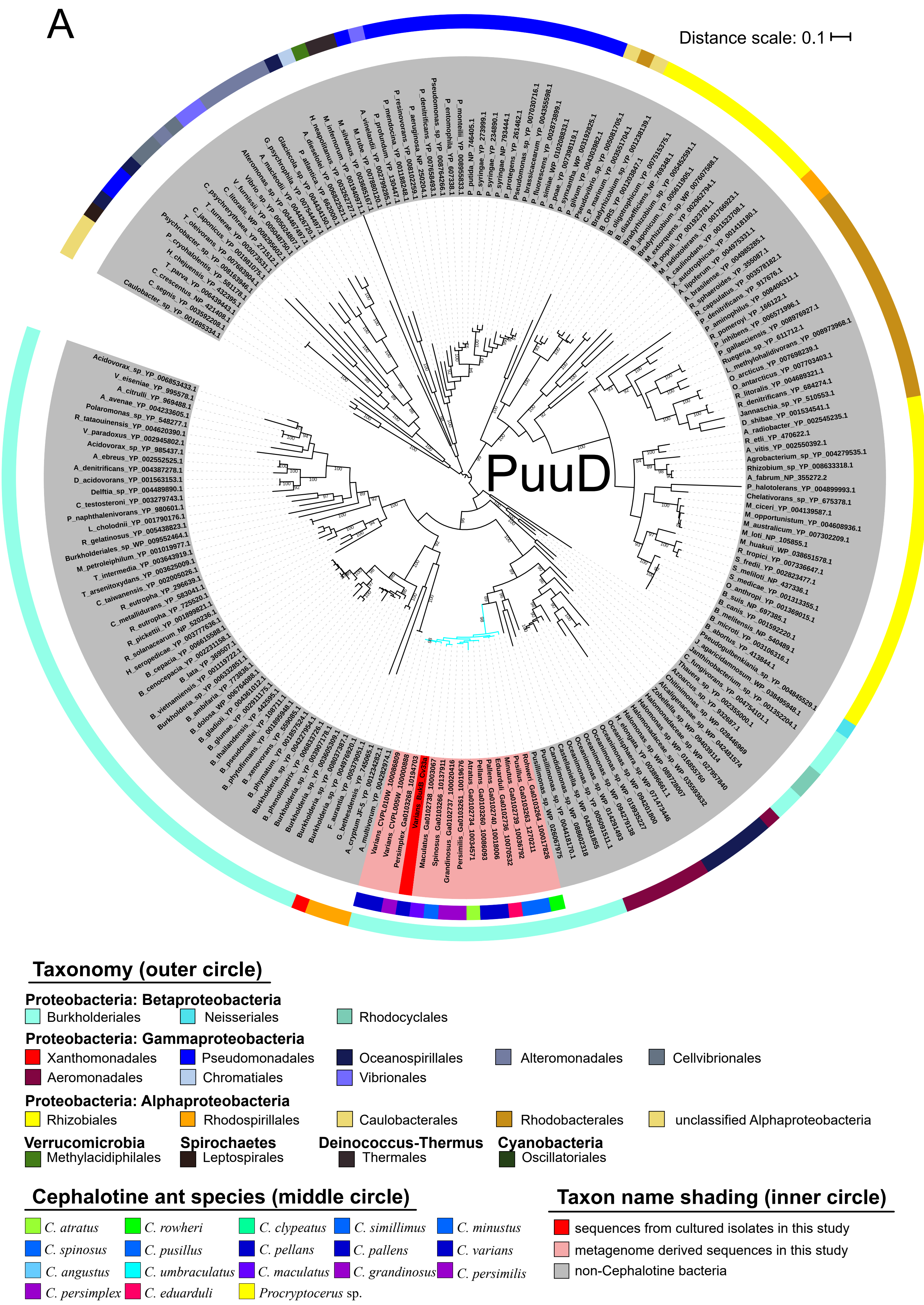
A
Distance scale: 0.1
PuuD
Taxonomy (outer circle)
Proteobacteria: Betaproteobacteria
Burkholderiales
Neisseriales
Rhodocyclales
Proteobacteria: Gammaproteobacteria
Xanthomonadales
Pseudomonadales
Oceanospirillales
Alteromonadales
Cellvibrionales
Aeromonadales
Chromatiales
Vibrionales
Proteobacteria: Alphaproteobacteria
Rhizobiales
Rhodospirillales
Caulobacterales
Rhodobacterales
unclassified Alphaproteobacteria
Verrucomicrobia
Methylacidiphilales
Spirochaetes
Leptospirales
Deinococcus-Thermus
Thermales
Cyanobacteria
Oscillatoriales
Cephalotine ant species (middle circle)
C. atratus
C. rowheri
C. clypeatus
C. simillimus
C. minustus
C. spinosus
C. pusillus
C. pellans
C. pallens
C. varians
C. angustus
C. umbraculatus
C. maculatus
C. grandinosus
C. persimilis
C. persimplex
C. eduarduli
Procryptocerus sp.
Taxon name shading (inner circle)
sequences from cultured isolates in this study
metagenome derived sequences in this study
non-Cephalotine bacteria

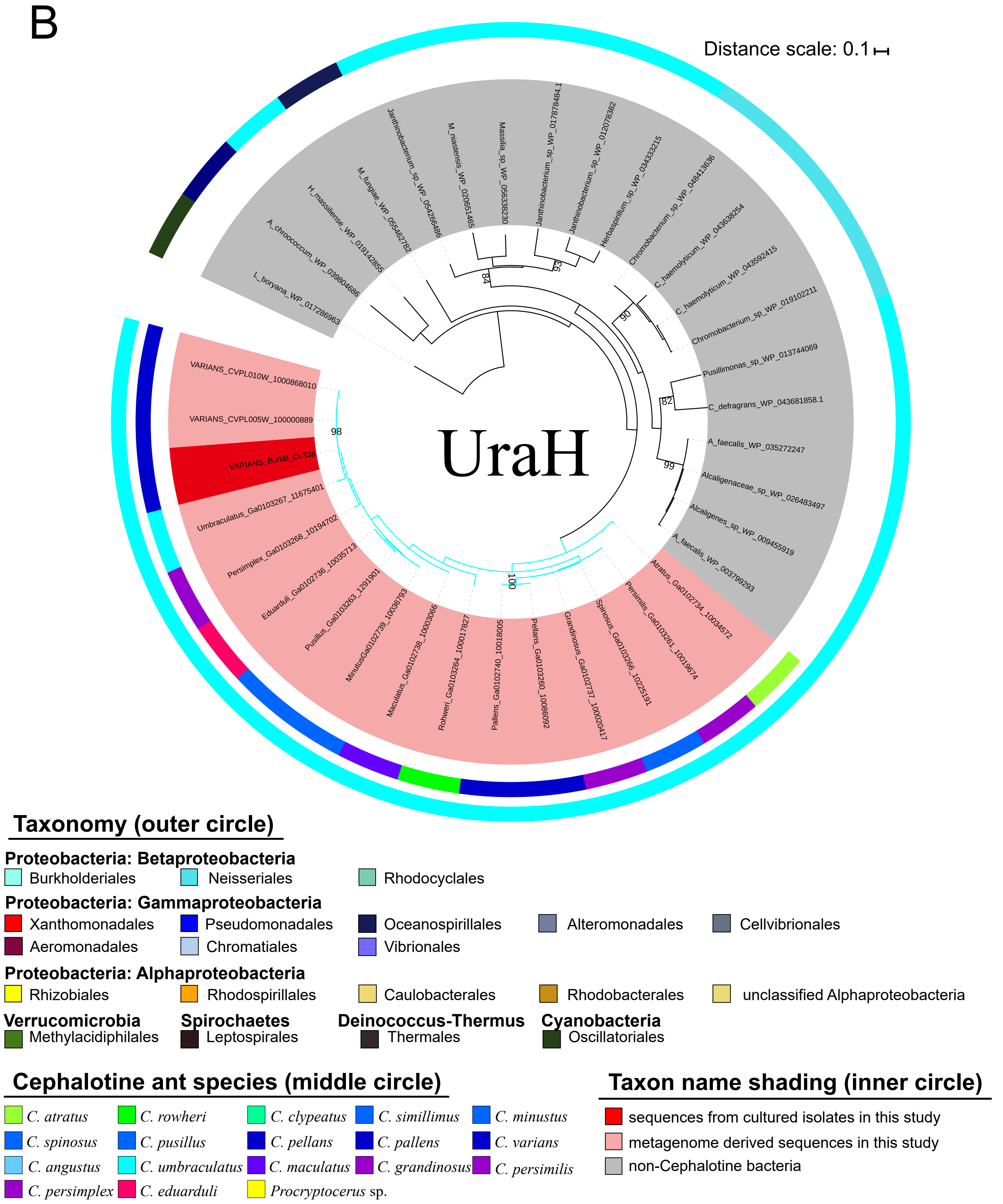

Supplementary Figure 11. Phylogeneties of symbiont PuuD and UraH proteins reveal patterns of convergent functional evolution among worker-associated gut bacteria. Phylogenies of PuuD proteins (A) and UraH proteins (B) based on sequences extracted from 18 *Cephalotes* metagenomes and top BLAST hits. Rooted maximum likelihood phylogeny reveals nearly all *Cephalotes*-associates come from *Cephalotes*-specific clades. Outer circle and branch colors: bacterial taxonomy. Middle circle colors: *Cephalotes* species groups. Inner circle: all red shading of taxon names reveals sequences coming from our metagenomic datasets, bright red shading of taxon names reveals cultured isolates and gray shading of taxon names represents bacteria found in hosts/habitats outside of Cephalotine ants.

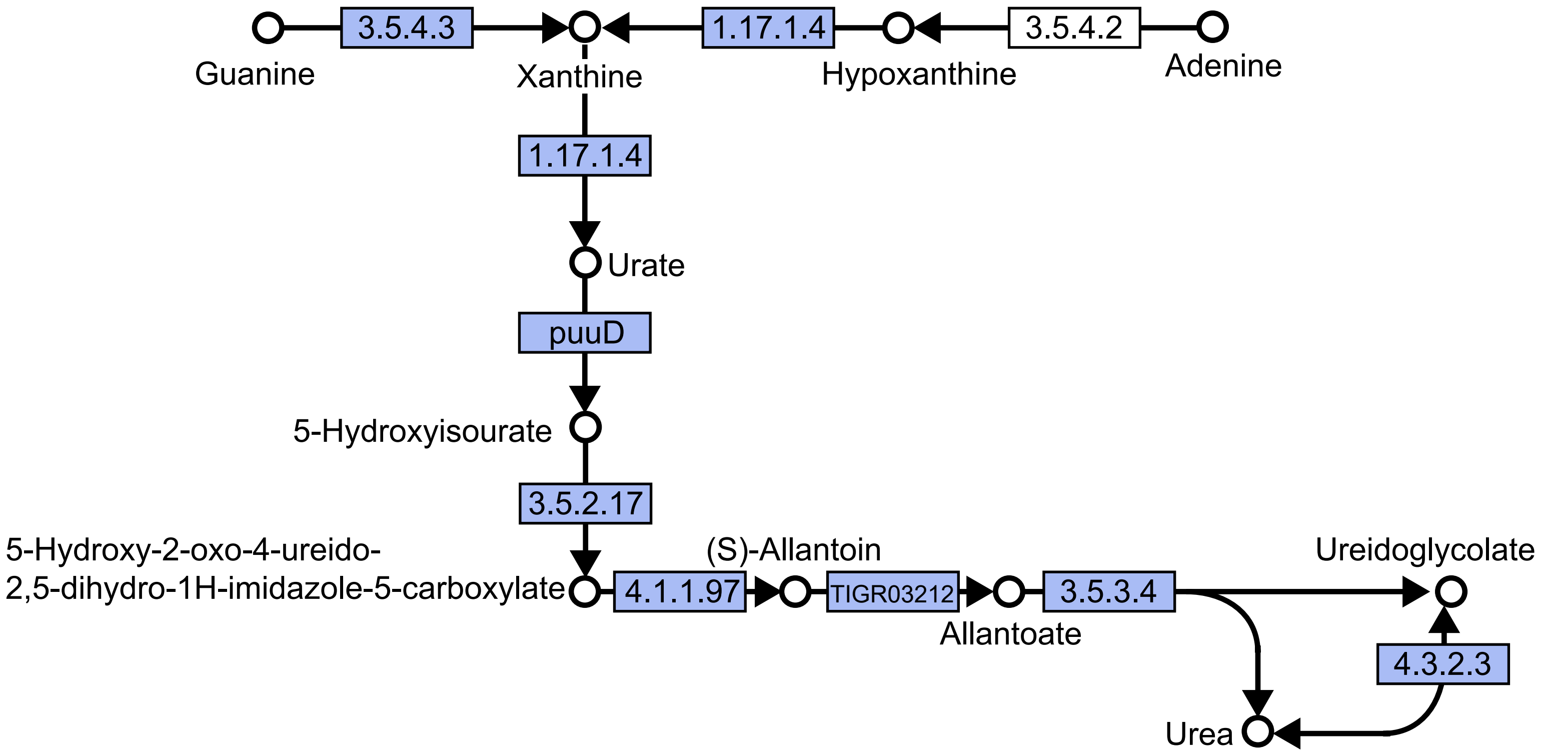

Supplementary Figure 12. *Cephalotes*' gut microbes encode purine deaminases, enabling the synthesis of uric acid and, eventually, urea from Guanine and Adenine. Shown with blue highlighted boxes are enzymes encoded by the Burkholderiales Cv33a strain, which can make urea from Guanine but potentially not Adenine.

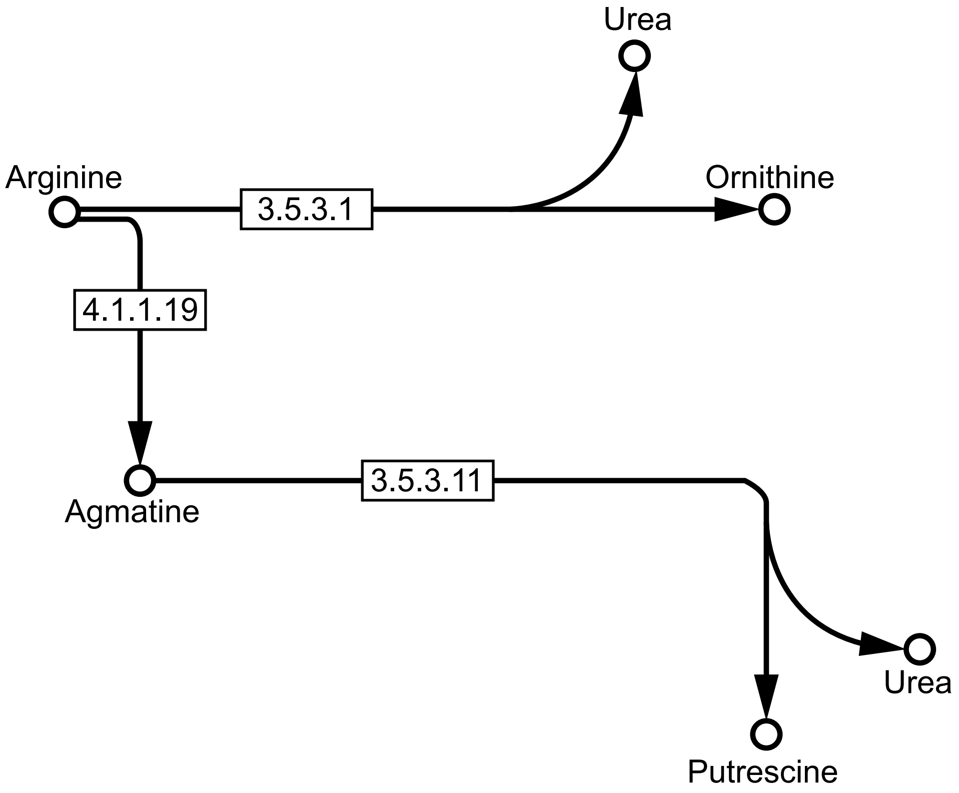

Supplementary Figure 13. Alternative mechanisms for N-recycling by gut symbionts of *Cephalotes* ants, through two distinct pathways converting arginine to urea.

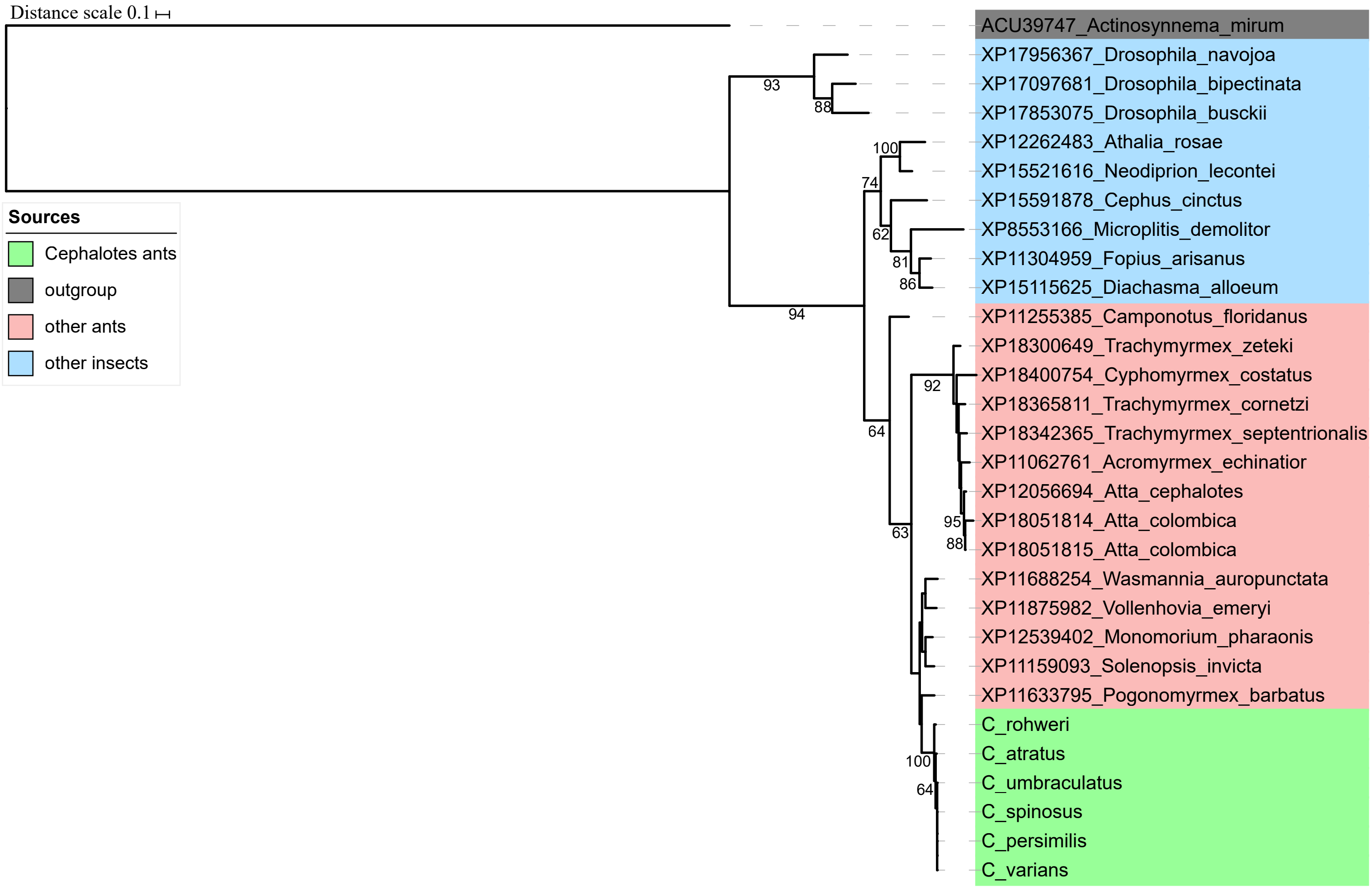

Supplementary Figure 14. Phylogeny of uricase amino acid sequences from metagenomes of *C. varians*. Maximum likelihood phylogeny reveals that uricase amino acid sequences in our metagenomic surveys form a *Cephalotes*-specific clade. The tree was rooted using *Actinosynnema mirum* as the outgroup. Clade colors represent the source from which the uricase coding sequences was derived.

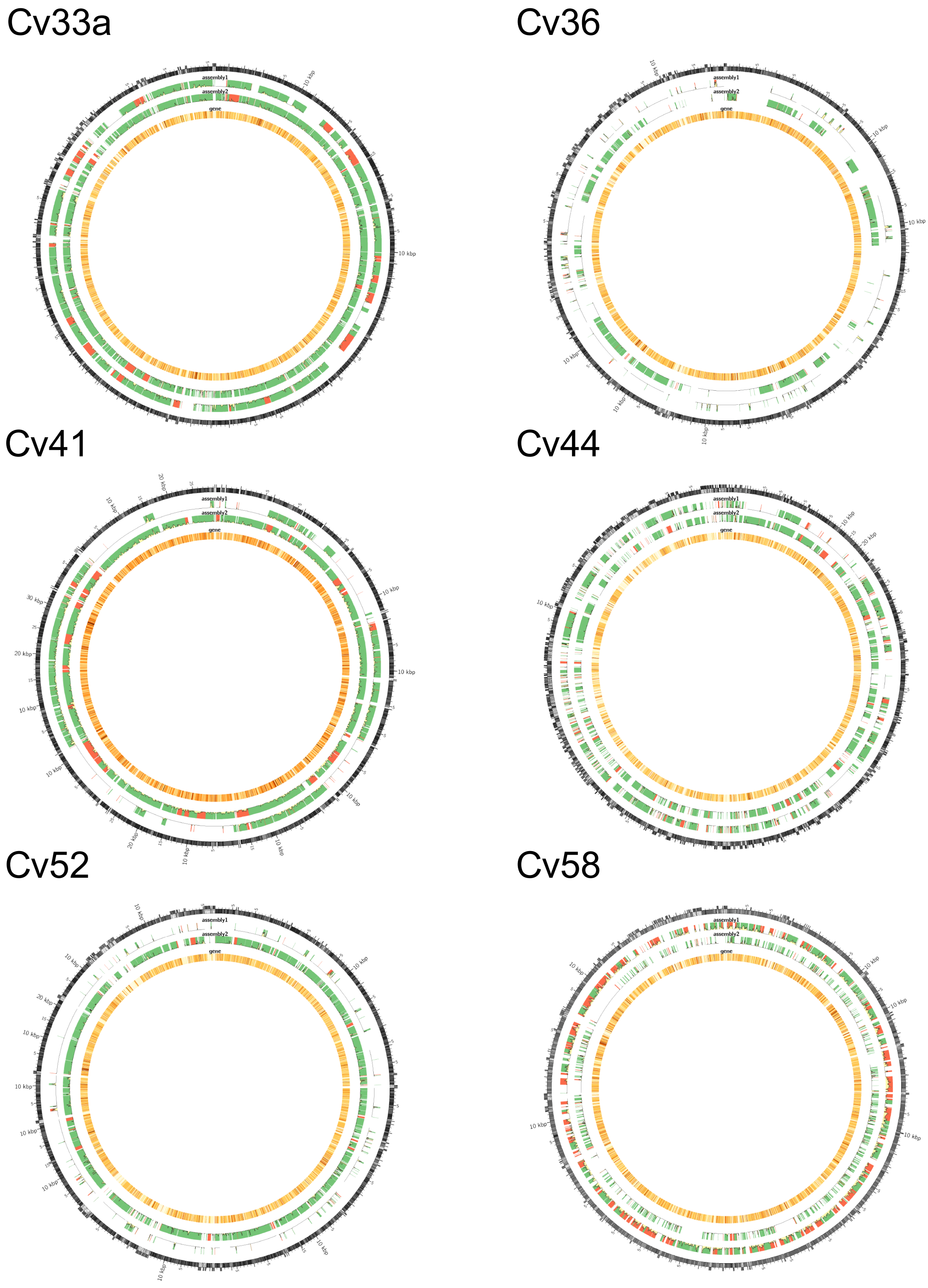

Supplementary Figure 15. Alignment of isolate genome assemblies with metagenome assemblies. Genome alignment against metagenome contigs shows the similarity of cultured isolates to genomes present in the *in vivo* gut community. Each circular genome visualization represents an isolate genome. The outermost ring shows GC%, while the innermost shows coding density of the isolate genome. Each of the two middle rings indicates alignment of scaffolds from *C. varians* metagenomes, from samples *C. varians* PL010 (inner) and *C. varians* PL005 (outer). For each sample, contigs aligning contiguously to the isolate genome reference are indicated by green blocks; contigs that align successfully but that are misassembled with respect to the reference isolate genome are indicated in red. Nucleotide mismatches between the reference and metagenome contigs are summarized by column charts within each band, with higher columns indicating moremismatches in that window.

Supplementary Table 1 Collection information for the ant colonies utilized in this study

Colony	Ant species	Collection locality	Latitude and Longitude	Date collected	Collector	Project for which each colony was used
YH001	*C. varians*	John Pennekamp Coral Reef State Park, Mangrove trail, Key Largo, Monroe Co., Florida, USA	25° 7' 25.38"N, 80° 24' 10.32"W	Aug. 30 ,2011	Yi Hu	Acetylene reduction assay
YH026	*C. varians*	Crocodile Lake Nat'l Wildlife Refuge, Key Largo, Monroe Co., Florida, USA	25° 11' 36.66"N, 80° 21' 25.26"W	Aug. 30 ,2011	Yi Hu	Acetylene reduction assay
CSM2266	*C. varians*	Crocodile Lake Nat'l Wildlife Refuge, Key Largo, Monroe Co., Florida, USA	25° 16' 45.84"N, 80° 20' 33.96"W	Aug.29, 2011	Corrie Moreau	Acetylene reduction assay
PL215A	*C. varians*	Little Hamaca Park, Key West, Monroe Co., Florida, USA	24° 33' 33.66"N, 81° 45' 41.76"W	Jan. 5, 2014	Piotr Lukasik	Feeding experiments with 15N-labelled urea
PL217	*C. varians*	Little Hamaca Park, Key West, Monroe Co., Florida, USA	24° 33' 33.66"N, 81° 45' 41.76"W	Jan. 5, 2014	Piotr Lukasik	Feeding experiments with 15N-labelled urea
PL231	*C. varians*	Road towards Long Beach, Big Pine Key, , Monroe Co., Florida, USA	24° 38' 28.57"N, 81° 20' 22.27"W	Jan. 5, 2014	Piotr Lukasik	Feeding experiments with 15N-labelled urea
PL207	*C. varians*	Little Hamaca Park, Key West, Monroe Co., Florida, USA	24° 33' 33.66"N, 81° 45' 41.76"W	Jan. 5, 2014	Piotr Lukasik	Feeding experiments with 13C-labelled glutamate
PL210	*C. varians*	Little Hamaca Park, Key West, Monroe Co., Florida, USA	24° 33' 33.66"N, 81° 45' 41.76"W	Jan. 5, 2014	Piotr Lukasik	Feeding experiments with 13C-labelled glutamate
PL216	*C. varians*	Little Hamaca Park, Key West, Monroe Co., Florida, USA	24° 33' 33.66"N, 81° 45' 41.76"W	Jan. 5, 2014	Piotr Lukasik	Feeding experiments with 13C-labelled glutamate
YH105	*C. varians*	Crocodile Lake Nat'l Wildlife Refuge, Key Largo, Monroe Co., Florida, USA	25°17'33.12"N, 80°18'19.74"W	Oct. 4, 2012	Yi Hu	Feeding experiments with 15N-labelled glutamate
YH115	*C. varians*	Crocodile Lake Nat'l Wildlife Refuge, Key Largo, Monroe Co., Florida, USA	25°17'2.64"N, 80°19'4.2"W	Oct. 4, 2012	Yi Hu	Feeding experiments with 15N-labelled glutamate
YH121	*C. varians*	Crocodile Lake Nat'l Wildlife Refuge, Key Largo, Monroe Co., Florida, USA	25°17'2.64"N, 80°19'4.2"W	Oct. 4, 2012	Yi Hu	Feeding experiments with 15N-labelled glutamate
PL005	*C. varians*	John Pennekamp Coral Reef State Park, mangrove trail, Key Largo, Monroe Co., Florida, USA	25° 7' 24.59"N, 80° 24' 11.88"W	Jun. 9, 2012	Piotr Lukasik	Metagenome sequencing
PL010	*C. varians*	Crocodile Lake Nat'l Wildlife Refuge, Tubby's Creek Bridge on Card Sound Rd., , Key Largo, Monroe Co., Florida, USA	25° 11' 36.66"N, 80° 21' 25.26"W	Jun. 11, 2012	Piotr Lukasik	Metagenome sequencing
JS-C-053	*C. angustus*	CICRA, Madre de Dios, Peru	12° 30' 0" S, 70° 5' 59.99" W	Jul. 20, 2010	Jon Sanders	Metagenome sequencing
JS-C-015	*C. atratus*	Uberlandia, Minas Gerais, Brazil	19° 6' 35.99" S, 48° 14' 24" W	Jun. 15, 2010	Jon Sanders	Metagenome sequencing
JS-C-011	*C. clypeatus*	Uberlandia, Minas Gerais, Brazil	19° 6' 35.99" S, 48° 14' 24" W	Jun. 10, 2010	Jon Sanders	Metagenome sequencing
JS-C-020	*C. eduarduli*	Uberlandia, Minas Gerais, Brazil	19° 6' 35.99" S, 48° 14' 24" W	Jun. 15, 2010	Jon Sanders	Metagenome sequencing
JS-C-001	*C. grandinosus*	Uberlandia, Minas Gerais, Brazil	19° 6' 35.99" S, 48° 14' 24" W	Jun. 5, 2010	Jon Sanders	Metagenome sequencing

Colony	Ant species	Collection locality	Latitude and Longitude	Date collected	Collector	Project for which each colony was used
JS-C-019	*C. maculatus*	Uberlandia, Minas Gerais, Brazil	19° 6' 35.99" S, 48° 14' 24" W	Jun. 15, 2010	Jon Sanders	Metagenome sequencing
JS-C-032	*C. minutus*	Uberlandia, Minas Gerais, Brazil	19° 6' 35.99" S, 48° 14' 24" W	Jun. 20, 2010	Jon Sanders	Metagenome sequencing
JS-C-025	*C. pallens*	Uberlandia, Minas Gerais, Brazil	19° 6' 35.99" S, 48° 14' 24" W	Jun. 17, 2010	Jon Sanders	Metagenome sequencing
JS-C-010	*C. pellans*	Uberlandia, Minas Gerais, Brazil	19° 6' 35.99" S, 48° 14' 24" W	Jun. 7, 2010	Jon Sanders	Metagenome sequencing
JS-C-018	*C. persimilis*	Uberlandia, Minas Gerais, Brazil	19° 6' 35.99" S, 48° 14' 24" W	Jun. 15, 2010	Jon Sanders	Metagenome sequencing
JS-C-054	*C. pusillus*	Uberlandia, Minas Gerais, Brazil	19° 6' 35.99" S, 48° 14' 24" W	Jul. 20, 2010	Jon Sanders	Metagenome sequencing
JS-C-012	*C. rohweri*	Tuscon, Arizona, USA	32° 12' 0" N, 110° 54' 0" W	Jun. 14, 2010	Jon Sanders	Metagenome sequencing
JS-C-063	*C. similimus*	CICRA, Madre de Dios, Peru	12° 30' 0" S, 70° 5' 59.99" W	Sep. 27, 2010	Jon Sanders	Metagenome sequencing
JS-C-045	*C. spinosus*	CICRA, Madre de Dios, Peru	12° 30' 0" S, 70° 5' 59.99" W	Jul. 1, 2010	Jon Sanders	Metagenome sequencing
JS-C-047	*C. umbraculatus*	CICRA, Madre de Dios, Peru	12° 30' 0" S, 70° 5' 59.99" W	Jul. 3, 2010	Jon Sanders	Metagenome sequencing
JS-C-050	*C. persimplex*	CICRA, Madre de Dios, Peru	12° 30' 0" S, 70° 5' 59.99" W	Jul. 11, 2010	Jon Sanders	Metagenome sequencing
YH106	*C. varians*	Crocodile Lake Nat'l Wildlife Refuge, Key Largo, Monroe Co., Florida, USA	25°17.552'N, 80°18.329'W	Oct. 4, 2012	Yi Hu	Genome sequencing for isolates CV41, CV36, CV44, CV33a, CV58, CV52
POW0550	*C. varians*	Key Largo, Monroe Co., Florida, USA	25° 10' 53" N, 80° 21' 45"W	Sep. 2016	Scott Powell	Genome sequencing for isolates POW0550W-166, POW0550W-2, POW0550W-160, POW0550W-89, POW0550W-4, POW0550W-97, POW0550W-103
CSM3487	*Procryptocerus pictipes*	French Guiana	3° 56' 2.04"N, 53° 7' 32.88"W	April, 2016	Corrie Moreau	Genome sequencing for isolates CSM3487-56, CSM3487-49, CSM3487-15
CSM3490	*Procryptocerus pictipes*	French Guiana	3° 56' 2.04" N, 53° 7' 32.88" W	April, 2016	Corrie Moreau	Genome sequencing for isolates CSM3490-51, CSM3490-52
JDR108-110	*C. texanus*	Sieppman Ranch, Cost, Gonzales, TX, USA	29° 27' 58.82"N, 97° 34' 2.32"W	Oct. 23, 2016	Jignasha Rana	Genome sequencing for isolates JDR108A-110-103, JDR108-110A-72, JDR108-110A-27, JDR108-110A-112, JDR108-110A-105, JDR108-110A-106, JDR108-110A-108, JDR108-110A-57.
JR038B	*C. texanus*	Floresville, TX, USA	27° 7' 54.13"N, 97° 47' 33.99" W	Jul. 22, 2015	Jacob Russell	Genome sequencing for isolates JR038B-95
SP57	*C. rohweri*	Tucson, AZ, USA	32° 13' 18.12" N, 110° 55' 35.4" W	July, 2015	Scott Powell	Genome sequencing for isolates SP57-223A, SP57-225
NA	*C. rohweri*	Tucson, AZ, USA	32° 13' 18.12" N, 110° 55' 35.4" W	2010	Scott Powell	Genome sequencing for isolates CAG34

Supplementary Table 2. Acetylene-reduction activity detected for *in vivo* bacterial communities of *C. varians* and information on colonies used in this study. Nitrogenase can reduce acetylene (C_2H_2) to ethylene (C_2H_4). No ethylene was detected in three ant colonies investigated in this study.

		YH001		YH026		CSM2266		control	
Caste and developmental	# of Workers	79		87		72		NA	
	# of Larvae	6		4		0		NA	
	# of Pupae	0		3		15		NA	
	# of Queens	0		0		1		NA	
Results of Acetylene-reduction assay	Sampling time (h)	C_2H_2 (ppm)	C_2H_4 (ppm)	C_2H_2 (ppm)	C_2H_4 (ppm)	C_2H_2 (ppm)	C_2H_4 (ppm)	C_2H_2 (ppm)	C_2H_4 (ppm)
	0h	22.8	0	27.3	0	27.7	0	13.3	0
	1h	11.7	0	8.8	0	11.5	0	12.2	0
	2h	12.7	0	15.9	0	10.6	0	12.9	0
	4h	20.5	0	18.7	0	18.9	0	21.1	0
	8h	24.7	0	13.6	0	22.4	0	26.6	0
	16h	29	0	19.4	0	25.8	0	26.2	0

Supplementary Table 3. Statistical results for heavy isotopic signal in hemolymph amino acids in three feeding experiments, including (a) ^{15}N-urea feeding experiment, (b) ^{13}C-glutamate feeding experiment and (c) ^{15}N-glutamate feeding experiment.

(a)

	**^{15}N-urea feeding experiment**	Arg	Ile	Leu	Lys	Met	Phe	Thr	Val	Ala	Asn	Asp	Cys	Glu	Gly	Pro	Tyr	Trp
	Shapiro-Wilk normality test	0.7324	0.4904	0.1323	0.0209	0.0293	0.8073	0.2587	0.2597	4.19E-05	0.0135	0.0023	1.97E-06	0.0039	0.0005	0.2089	0.0079	NA
Parametric test	Effect of diet on the ^{15}N signal of amino acids in different dietary groups revealed by an ANOVA test	**<2e-16**	**<2e-16**	**<2e-16**	NA	NA	**<2e-16**	**<2e-16**	**<2e-16**	NA	NA	NA	NA	NA	NA	**<2e-16**	NA	NA
	Pairwise comparison between ^{15}N-urea vs ^{15}N-urea-antibiotics using post hoc Tukey HSD test	**1.54E-11**	**4.36E-14**	**4.7E-14**	NA	NA	**4.36E-14**	**4.36E-14**	**4.5E-14**	NA	NA	NA	NA	NA	NA	**1.92E-14**	NA	NA
	Pairwise comparison between ^{15}N-urea vs ^{14}N-urea using post hoc Tukey HSD test	**4.36E-14**	**4.36E-14**	**4.43E-14**	NA	NA	**4.36E-14**	**4.36E-14**	**4.36E-14**	NA	NA	NA	NA	NA	NA	**1.92E-14**	NA	NA
	Pairwise comparison between ^{14}N-urea vs ^{15}N-urea-antibiotics using post hoc Tukey HSD test	**1.02E-10**	0.6717	0.1175	NA	NA	**0.0039**	0.0933	**0.0195**	NA	NA	NA	NA	NA	NA	**5.17E-11**	NA	NA
Non-parametric test	Effect of diet on the ^{15}N signal of amino acids in different dietary groups revealed by a Kruskal Wallis test	NA	NA	NA	**0.0003**	**0.0101**	NA	NA	NA	**0.0001**	0.1009	**3.54E-05**	**0.0012**	**3.48E-05**	**3.51E-05**	NA	**0.0001**	NA
	Pairwise comparison between ^{15}N-urea vs ^{15}N-urea-antibiotics using post hoc Mann-Whitney test	NA	NA	NA	**0.0027**	**0.0410**	NA	NA	NA	**0.03124**	1.0000	**0.0028**	**0.0058**	**0.0028**	**0.0028**	NA	**0.0028**	NA
	Pairwise comparison between ^{15}N-urea vs ^{14}N-urea using post hoc Mann-Whitney test	NA	NA	NA	**0.0027**	**0.0210**	NA	NA	NA	**0.00093**	1.0000	**0.0028**	**0.0058**	**0.0027**	**0.0027**	NA	**0.0028**	NA
	Pairwise comparison between ^{14}N-urea vs ^{15}N-urea-antibiotics using post hoc Mann-Whitney test	NA	NA	NA	0.4541	0.2340	NA	NA	NA	**0.0005**	0.0720	**0.0028**	1.0000	**0.0027**	**0.0027**	NA	0.0712	NA

(b)

	^{13}C-glutamate feeding experiment	Arg	Ile	Leu	Lys	Met	Phe	Thr	Val	Ala	Asn	Asp	Cys	Glu	Gly	Pro	Tyr	Trp
	Shapiro-Wilk normality test	0.2098	0.7948	0.8147	1.50E-05	0.0003	0.6839	0.0034	0.0545	0.4205	0.0069	0.3938	0.6566	0.0242	0.9524	0.0097	0.0002	NA
Parametric test	Effect of diet on the ^{13}C signal of amino acids in different dietary groups revealed by an ANOVA test	**2.85E-09**	**0.0089**	**0.0002**	NA	NA	**2.10E-05**	NA	**0.0158**	**2.11E-07**	NA	**0.0245**	0.2310	NA	0.6590	NA	NA	NA
	Pairwise comparison between ^{13}C-glu vs ^{13}C-glu-antibiotics using post hoc Tukey HSD test	0.7973	**0.0147**	**0.0004**	NA	NA	**0.0709**	NA	0.2049	0.2195	NA	0.3048	0.7396	NA	0.9984	NA	NA	NA
	Pairwise comparison between ^{13}C-glu vs ^{12}C-glu using post hoc Tukey HSD test	**1.33E-08**	**0.0219**	**0.0013**	NA	NA	**1.46E-05**	NA	**0.0118**	**0.0001**	NA	0.3514	0.2063	NA	0.7305	NA	NA	NA
	Pairwise comparison between ^{12}C-glu vs ^{13}C-glu-antibiotics using post hoc Tukey HSD test	**2.73E-08**	0.9953	0.9389	NA	NA	**0.0026**	NA	0.3112	**9.00E-07**	NA	**0.0188**	0.5448	NA	0.6845	NA	NA	NA
Non-parametric test	Effect of diet on the ^{13}C signal of amino acids in different dietary groups revealed by a Kruskal Wallis test	NA	NA	NA	**0.0225**	0.0862	NA	**0.0005**	NA	NA	0.9497	NA	NA	**0.0004**	NA	**0.0002**	**0.0004**	NA
	Pairwise comparison between ^{13}C-glu vs ^{13}C-glu-antibiotics using post hoc Mann-Whitney test	NA	NA	NA	0.0900	0.8000	NA	**0.0029**	NA	NA	1.0000	NA	NA	1.0000	NA	0.2487	**0.0169**	NA
	Pairwise comparison between ^{13}C-glu vs ^{12}C-glu using post hoc Mann-Whitney test	NA	NA	NA	**0.0410**	0.3400	NA	**0.0025**	NA	NA	1.0000	NA	NA	**0.0018**	NA	**0.0027**	**0.0025**	NA
	Pairwise comparison between ^{12}C-glu vs ^{13}C-glu-antibiotics using post hoc Mann-Whitney test	NA	NA	NA	**>0.05**	0.2000	NA	**>0.05**	NA	NA	1.0000	NA	NA	**0.0018**	NA	**0.0018**	0.0890	NA

(c)

	^{15}N-glutamate feeding experiment	Arg	Ile	Leu	Lys	Met	Phe	Thr	Val	Ala	Asn	Asp	Cys	Glu	Gly	Pro	Tyr	Trp
	Shapiro-Wilk normality test	NA	0.2969	0.8673	0.8064	0.0002	0.0074	0.3081	0.0390	0.3012	NA	0.0851	NA	0.2041	0.0019	0.0642	NA	0.7195
Parametric test	Effect of diet on the ^{15}N signal of amino acids in different dietary groups revealed by an ANOVA test	NA	0.0794	0.0896	**0.0006**	NA	NA	**0.0381**	NA	**0.0012**	NA	**0.0052**	NA	**0.0041**	NA	**0.0086**	NA	**0.0010**
	Pairwise comparison between ^{15}N-glu vs ^{15}N-glu-antibiotics using post hoc Tukey HSD test	NA	0.0964	0.1015	0.9187	NA	NA	0.1942	NA	0.9999	NA	0.1925	NA	0.5320	NA	0.2055	NA	0.7645
	Pairwise comparison between ^{15}N-glu vs ^{14}N-glu using post hoc Tukey HSD test	NA	0.1869	0.2238	**0.0007**	NA	NA	**0.0364**	NA	**0.0017**	NA	**0.0039**	NA	**0.0033**	NA	**0.0066**	NA	**0.0010**
	Pairwise comparison between ^{14}N-glu vs ^{15}N-glu-antibiotics using post hoc Tukey HSD test	NA	0.7376	0.6991	**0.0067**	NA	NA	0.8862	NA	**0.0076**	NA	0.3329	NA	0.0911	NA	0.4300	NA	**0.0160**
Non-parametric test	Effect of diet on the ^{15}N signal of amino acids in different dietary groups revealed by a Kruskal Wallis test	NA	NA	NA	NA	0.0608	**0.0057**	NA	**0.0246**	NA	NA	NA	NA	NA	0.4947	NA	NA	NA
	Pairwise comparison between ^{15}N-glu vs ^{15}N-glu-antibiotics using post hoc Mann-Whitney test	NA	NA	NA	NA	0.1600	**0.0450**	NA	0.1500	NA	NA	NA	NA	NA	1.0000	NA	NA	NA
	Pairwise comparison between ^{15}N-glu vs ^{14}N-glu using post hoc Mann-Whitney test	NA	NA	NA	NA	0.2000	**0.0130**	NA	**0.0600**	NA	NA	NA	NA	NA	1.0000	NA	NA	NA
	Pairwise comparison between ^{14}N-glu vs ^{15}N-glu-antibiotics using post hoc Mann-Whitney test	NA	NA	NA	NA	1.0000	1.0000	NA	1.0000	NA	NA	NA	NA	NA	1.0000	NA	NA	NA

Supplementary Table 4. Assembly statistics of metagenomic data.

Ant species	Reads numbers	Total length Mbp	Total >= 1 Kbp scaffold length (Mbp)	Scaffold number	Scaffold number (>=1Kbp)	N50 for scaffold length [bp]	GC% mean	Read coverage mean	IMG project ID
***C. varians* PL005W**	40,804,316	184.98	142.43	281,884	129,829	1,074	39.18%	16.60	Gp0095985
***C. varians* PL010W**	135,924,092	436.39	340.3	647,079	91,083	10,750	41.95%	41.75	Gp0095983
C. angustus	3,534,316	21.5	8.22	30,858	3,531	1,118	50.80%	6.79	Gp0125961
C. atratus	34,205,408	218.88	141.37	198,281	67,767	1,488	38.53%	9.20	Gp0125962
C. clypeatus	24,093,876	104.18	41.7	135,743	16,952	1,031	37.42%	8.62	Gp0125963
C. eduarduli	22,505,270	82.63	37.38	100,267	12,793	1,259	39.99%	9.53	Gp0125964
C. grandinosus	37,871,910	156.24	60.81	220,221	22,090	1,215	39.68%	10.06	Gp0125967
C. maculatus	40,739,970	144.43	42.17	230,315	18,066	921	39.60%	7.52	Gp0125968
C. minutus	31,576,424	115.64	37.69	180,629	15,101	1,038	38.38%	8.10	Gp0125969
C. pallens	28,073,250	148.44	55.05	211,667	21,214	1,095	39.56%	7.92	Gp0125970
C. pellans	33,836,572	142.82	43.13	224,021	18,268	934	38.32%	8.51	Gp0126569
C. persimilis	28,044,242	106.24	44.84	133,984	19,037	1,077	40.41%	10.09	Gp0126571
C. persimplex	40,440,804	231.49	102.43	291,903	53,617	1,090	37.94%	10.14	Gp0126580
C. pusillus	18,252,188	61.89	24.03	93,263	9,713	1,389	42.35%	6.67	Gp0126572
C. rohweri	58,943,942	305.84	271.58	142,961	78,950	3,982	40.47%	22.40	Gp0126573
C. simillimus	30,304,070	92.55	37.04	122,379	16,294	1,030	37.75%	13.09	Gp0126574
C.spinosus	19,456,806	94.81	41	117,242	13,728	1,206	38.79%	8.66	Gp0126575
C. umbraculatus	37,344,984	256.42	193.69	191,339	84,591	1,984	37.76%	14.53	Gp0126577

Supplementary Table 5. Assembly statistics of genomes and cultivation conditions of cultured bacteria.

Genome Name	Total length [bp]	Scaffold number	N50 [bp]	Protein CDS	GC %	IMG Project ID	Ant host	Sequencing technique	Cultivation media and conditions
Cephaloticoccus primus **Cag34**	2353466	23	207028	1871	62.43%	Gp0154034	*C. rohweri*	Illumina Hiseq	Those bacteria were cultivated and maintained in tryptic soy agar or tryptic soy broth under an atmosphere of normal air supplemented with 1% carbon dioxide in a CO_2-controlled water-jacketed incubator at 25 ℃.
Cephaloticoccus capnophilus **Cv41**	2094663	34	169871	1683	59.29%	Gp0110136	*C. varians*	Illumina Hiseq	
**Rhizobiales sp. JR021-5**	1943462	1	1943462	1586	57.28%	Gp0155340	*C. varians*	PacBio	
**Xanthomonadales sp. Cag60**	3424135	179	44406	3201	56.17%	Gp0127963	*C. rohweri*	Illumina Hiseq	
Ventosimonas gracilis **Cv58**	2623100	221	58779	2612	53.40%	Gp0110146	*C. varians*	Illumina Hiseq	
Ventosimonas **sp. Cag26**	2729219	92	79155	2684	54.82%	Gp0154031	*C. rohweri*	Illumina Hiseq	
Ventosimonas **sp. Cag27**	2719975	89	79155	2668	54.89%	Gp0154032	*C. rohweri*	Illumina Hiseq	
Ventosimonas **sp. Cag320**	2825500	94	108424	2804	55.67%	Gp0154033	*C. rohweri*	Illumina Hiseq	
**Burkholderiales sp. Cag20**	3101960	121	69296	3051	58.26%	Gp0154021	*C. rohweri*	Illumina Hiseq	
**Burkholderiales sp. Cag25**	3191349	112	142682	3073	58.13%	Gp0154030	*C. rohweri*	Illumina Hiseq	
**Burkholderiales sp. Cv44**	3159338	601	33742	3318	60.40%	Gp0110143	*C. varians*	Illumina Hiseq	
**Burkholderiales sp. Cv33a**	2469676	150	48592	2380	58.91%	Gp0110144	*C. varians*	Illumina Hiseq	
**Burkholderiales sp. Cv36**	2921795	234	54338	2858	59.95%	Gp0110137	*C. varians*	Illumina Hiseq	
**Burkholderiales sp. Cv52**	2935707	203	93689	2820	60.36%	Gp0110145	*C. varians*	Illumina Hiseq	

Supplementary Table 6. Summary of strain-level binning for gut metagenomes from *C. varians* workers in colony PL010.

Bin ID	Bacterial order	total length	scaffold numbers	N50 [bp]	GC %		Read depth		complete%	redundancy%
					average	SD	average	SD		
**Bin_12**	Burkholderiales	2296599	112	29329	56.24	1.80	197.79	9.12	98.87%	4.82%
**Bin_5_1**	Burkholderiales	1698366	136	15370	63.64	1.36	138.20	12.30	89.69%	3.42%
**Bin_3_1**	Burkholderiales	1649427	143	12790	60.93	1.23	150.59	8.94	87.79%	3.75%
**Bin_6_3**	Rhizobiales	2010514	62	45267	49.26	0.89	23.18	1.40	97.39%	3.64%
**Bin_13**	Rhizobiales	2164522	101	30246	48.56	1.24	19.05	3.69	96.12%	5.00%
**Bin_6_2**	Rhizobiales	2013369	92	28841	50.56	0.89	37.39	1.55	95.41%	3.46%
**Bin_11**	Sphingobacteriales	2906493	126	31687	54.93	2.30	68.79	5.69	96.76%	5.83%
**Bin_16**	Flavobacteriales	1851657	87	30302	34.69	1.41	122.10	16.36	96.62%	1.81%
**Bin_7_1**	Opitutales	2035389	45	72092	60.46	1.72	1966.13	115.78	96.40%	2.72%
**Bin_14**	Xanthomonadales	1990584	84	33484	49.76	2.05	1505.15	26.21	94.58%	4.38%
**Bin_17**	Campylobacterales	1490882	63	33424	27.66	1.21	14.71	6.99	90.69%	2.10%

Supplementary Methods

Assessing N-fixation

To measure N-fixation capacities for *Cephalotes* associated microbes we performed acetylene reduction assays, in which conversion of acetylene (C_2H_2) to ethylene (C_2H_4) is used as evidence for active nitrogenase enzymes[1, 2]. Three colonies of *C. varians* were collected (by CSM or YH) from mangrove trees in the Florida Keys (**Supplementary Table 1**). After excavation in the field, we immediately placed all available workers (and larvae, pupae or queens, when present: **Supplementary Table 2**) into 10 ml gas tight syringes (Vici Precision Sampling Inc, Baton Rouge, LA, USA). An empty syringe was used as a control. Two milliliters of air in these four syringes were removed and two milliliters of acetylene were added to the syringe, resulting in a final atmosphere of 20% acetylene. A 1ml air mixture sample from each syringe was injected in a 3ml Exetainer tube at 0, 1, 2, 4, 8, 16 hours. Acetylene and ethylene concentrations were then quantified using a gas chromatography-flame ionization detector (GC-FID, HP6890 series, Agilent Technologies, Inc., E.&E.S. Analytical Instrumentation of University of Pennsylvania).

Feeding experiments with ^{15}N-labeled urea and ^{13}C/^{15}N-labeled glutamate

Nine colonies of *C. varians* collected from the Florida Keys (**Supplementary Table 1**) were reared on a holidic artificial diet[3] and 50% honey water at 25°C under a daily light:dark cycle of 14:10 until use in the feeding experiment. Fresh diet was provided roughly every two days. All adult workers were subjected to a water-only starvation period of three days prior to the start of experiments.

In the feeding experiment with ^{15}N-labeled urea, workers from each of three colonies were split into two treatment groups. In the first treatment, workers were subjected to antibiotic feeding

to remove their gut bacteria through rearing on 30% (weight/volume) sucrose water containing 0.01% of each Tetracycline, Rifampicin, and Kanamycin. Untreated workers from the second treatment group consumed only 30% sucrose water. After the three week pre-trial period, the antibiotic-treatment groups were provided with 30% sucrose water with the same antibiotic mixture, in addition to 1% (weight/volume) ^{15}N-labeled urea (Sigma-Aldrich, St Louis, MO). Untreated ants were further split into subgroupings, with half being reared upon 30% sucrose water with 1% (w/v) ^{15}N-labeled urea, and the other half consuming 30% sucrose water containing 1% (w/v) unlabeled (i.e. mostly ^{14}N) urea.

The same experimental design was applied to three colonies in an experiment with ^{13}C-labeled glutamate (and unlabeled control) and to three additional colonies under a ^{15}N-labeled glutamate treatment (with an unlabeled control). Workers from each colony were divided into two groups, with the first group of workers being fed a holidic artificial diet[3] containing 0.01% of each Tetracycline, Rifampicin, and Kanamycin, and the second group consuming a holidic artificial diet for three weeks (the pre-trial period). At that point, antibiotic-treatment groups were then switched to the modified holidic diet (trial period), with only non-essential amino acids and the total amino acid concentration the same as the complete holidic diet, plus glutamate containing either isotope label, in addition to the same antibiotic mixture. At the same time, those from the treatment without antibiotics were split into two groups, with one set consuming the same diet as their antibiotic-treated counterparts, with either a standard isotope ratio (treatment 2) or with glutamate containing the heavy label (treatment 3). No antibiotics were added to these latter diets.

After four to five weeks of feeding during the trial period, ant hemolymph was extracted from surviving workers. Hemolymph was harvested from decapitated ants using borosilicate glass

needles pulled from microcapillary tubes (1/0.58 OD/ID mm, World Precision Instruments, Sarasota, FL) to capture droplets exuding from the posterior opening of the head capsule and from the anterior opening of the mesosoma. Depending on the number of ants available, there were two or three replicates for each colony and treatment, each consisting of pooled hemolymph from 3-10 workers (see **Supplementary Data 6** for details). Hemolymph was added to 10ul of molecular grade water, and samples frozen at -80°C immediately after collection.

Worker survival curves for the ^{13}C and ^{15}N experiments (trial periods) were plotted and data were analyzed using Cox regression analysis.

qPCR and amplicon 16S rRNA sequencing to estimate antibiotic efficacy

Quantative PCR with universal 16S rRNA primers was used to confirm that the antibiotic treatments drastically reduced bacterial loads in gut communities of *C. varians* (**Supplementary Fig. 2**). Gasters of those ants were used for DNA extraction and all other DNA isolation procedures were the same as mentioned below. 16S rRNA gene copy concentration was estimated using qPCR with PerfeCTa SYBR Green FastMix (Quanta Biosciences, Gaithersburg, MD, USA) and eubacterial primers 515F (5'-GTGCCAGCMGCCGCGGTAA-3') and 806R (5'-GGACTACHVGGGTWTCTAAT-3') at 200 nM each, on a CFX96 Touch™ Real-Time PCR Detection System (Bio-Rad, Hercules, CA, USA). The PCR program consisted of initial denaturation at 94°C for 3 minutes; 40 cycles of 94°C for 45s, 50°C for 60s, 72°C for 60s, and plate read at the end of the extension step. Melting curve analysis was applied at the end of these 40 cycles, with temperatures rising from 55°C to 95°C with 0.5°C increments and plate reads after 5s incubation at each temperature. A ten-fold dilution series of a DNA sample extracted from ten pooled guts from *C. varians*

workers was used to build the standard curve for estimating relative bacterial abundance in ants under different dietary treatments. Four biological replicates per dietary treatment in each colony were chosen for quantitative PCR. Three technical replicates per standard curve sample and two technical replicates per biological replicate were performed for each dietary experiment. The relative bacterial abundance was determined by dividing bacterial 16S rRNA copy number estimates by one tenth of the total amount of bacterial 16S rRNA copy number estimates of the pooled gut DNA sample used for constructing the standard curve. All relative bacterial abundance data (including eight biological replicates for each dietary treatment in each colony, except six biological replicates for 15N-glutamate treatment in colony YH105) were checked for normality by Shapiro-Wilk *W*-test. Normal data were compared using one-way ANOVA with dietary treatment as a factor and relative bacterial abundance as dependent variables, followed by Tukey's post-hoc tests. Non-normal data were analyzed by Kruskal-Wallis tests followed by multiple pairwise comparisons using the Wilcoxon rank sum test. All statistical tests were performed using R version 3.3.2.

DNA samples from individual workers in the ^{15}N glutamate experiment were sent to Argonne National Laboratory for Illumina amplicon sequencing of the V4 region of bacterial 16S rRNA. Analyses of sequences proceeded using previously published quality control and filtering protocols[4]. A 97% OTU table was generated (**Supplementary Data 7**), and the average relative abundance of each OTU was obtained across ants from the same treatment. Averages were then plotted in conjunction with qPCR data from the same treatment (**Supplementary Fig. 2**), showing how antibiotics had altered the composition in addition to the quantities of gut microbiota.

Amino acid analysis from ant hemolymph by gas-chromatography-mass spectrometry (GC-

MS)

Enrichment of amino acids in ant hemolymph was measured at the Metabolic Tracer Resource at the University of Pennsylvania. Approximately 5 ul of each hemolymph-water mixture was acidified with 1 ml of 1N acetic acid and run over AG 50W-X8 cation exchange resin. Resin was washed three times with milli-Q water and free amino acids eluted using 3N ammonium hydroxide. Samples were dried in a rotary vacuum evaporator and amino acids converted to their heptafluorobutyryl isobutyl ester derivatives[5]. Derivatized amino acids were injected onto an Agilent 7890A/5975C Series gas chromatograph/mass spectrometer (GC/MS) (Agilent Technologies, Santa Clara, CA) operated in the negative chemical ionization mode and separated using a DB5-MS column. The injection port temperature was 250°C. The GC column temperature was maintained at 80°C for 1 minute, increased to 150°C (10°C /min) and then to 300°C (20°C /min). It was then held at 300°C for 1 minute. Amino acid peaks were identified by retention time, which was confirmed using purified standards. Peaks that could not be definitively identified were not measured.

Abundance data of $^{15}N/^{13}C$-labeled essential amino acids in ant hemolymph samples were transformed with a logit transformation (ln(p/(1-p))) before statistical analysis. All logit transformed data were checked for normality by Shapiro-Wilk *W*-test. Normal data were compared using one-way ANOVA with dietary treatment as a factor and levels of ^{15}N or ^{13}C-labeled amino acids as dependent variables, followed by Tukey's post-hoc tests. Non-normal data were analyzed by Kruskal-Wallis tests followed by multiple pairwise comparisons using the Wilcoxon rank sum test (see **Supplementary Table 3**). All statistical tests were performed using R version 3.3.2.

DNA preparation for* C. varians *metagenomics

Ten adult *C. varians* workers from each of two colonies in the Florida Keys were used to create two DNA pools for metagenome sequencing. Adult workers were washed in 70% ethanol and sterile water before dissection. Ant guts were dissected with sterile forceps under a compound light microscope. Between each individual dissection, forceps were rinsed with a 6% bleach solution and then with sterile water. The dissected mid- and hind- guts were individually immersed in 180 µL enzymatic lysis buffer containing lysozyme (20 mg/ml). After grinding with sterile pestles, samples were incubated for 30 min at 37°C. Extractions then proceeded according to the protocol for gram-positive bacteria with the Qiagen DNeasy Kit (Qiagen, Valencia, CA). Pooled genomic DNA from the guts of ten workers per colony was used as source material for the two Illumina HiSeq metagenome libraries (colony PL005; colony PL010).

DNA extraction from non-*C. varians *ants for metagenomics

DNA from dissected guts of *Cephalotes* ants other than *C. varians* was extracted according to the protocol of Sanders *et al* 2014[6], using pools of 10 dissected guts per colony (as opposed to single guts, as for *C. varians*). Briefly, dissected guts preserved in RNAlater were diluted ~1:1 in sterile water (to decrease solution density and dissolve any precipitated salts), and spun to pellet the biological material. The supernatant was removed and replaced with lysis buffer TLS-C (MPBio, inc), then vortexed to resuspend. Resuspended material was lysed with Phenol:Chloroform:Isoamyl alchohol (pH 8) and sterile beads (Lysis Matrix A, MPBio) on a MPBio FastPrep-20 bead beater. The aqueous phase was then column-purified through Qiagen DNeasy Blood and Tissue extraction columns, and concentrated by isopropanol precipitation. Full methodological details are published elsewhere[6].

DNA extraction from cultured bacteria

High molecular weight DNA from cultured bacteria isolated from *C. varians* and *C. rohweri* was extracted using Qiagen Genomic Tip 20/G columns, following the manufacturer's recommendations for bacterial cultures.

Genome and metagenome sequencing, assembly and annotation

For shotgun sequencing of metagenomes of *C. varians* and isolates derived from *C. varians* and *C. rohweri*, DNA was sheared to 400bp using a Covaris S220 sonicator. Sheared DNA was end-repaired and ligated to indexed Illumina-compatible sequencing adapters (Bioo Scientific, Inc) using the KAPA low-throughput Illumina-compatible library preparation kit (KAPA biosystems, Inc). Fragments of the two prepared libraries were size selected using double-ended SPRI bead-based size selection following the KAPA protocol. After this selection, libraries were amplified for six cycles using KAPA high-fidelity polymerase and then checked for quality using an Agilent Bioanalyzer. The two prepared libraries as well as two from *Cephalotes* larval samples were pooled with other indexed samples, combining for an estimated 40% of the total molar fraction in the Illumina sequencing lane, and then sequenced at the Harvard Biopolymers Facility using paired-end 150 bp reads on an Illumina HiSeq 2500 instrument.

Sequence libraries for non-*C. varians*-derived metagenomes and isolates were prepared using the same Covaris shearing step as above, but on an Apollo 324 automated library preparation robot using the PrepX ILM DNA kit (IntegenX, Inc) following manufacturer’s recommendations. These libraries were PCR-amplified using the same protocol as above, and amplified libraries were size-selected using the double-ended SPRI bead-based size selection protocol on the Apollo 324 instrument. The resulting libraries were sequenced using paired-end 100bp chemistry on an Illumina HiSeq 2000 instrument.

Metagenome sequences were trimmed for quality and adapters using Trimmomatic[7]. The quality trimmed reads were then combined and assembled with IDBA-UD 1.1.1 using k values of 20, 40, 60, 80, and 100[8]. The assembled data were run through QUAST[9] to calculate assembly statistics (**Supplementary Table 4**).

Scaffolds of 18 metagenomes and 14 isolates, and coverage information of metagenomic scaffolds were uploaded to the Integrated Microbial Genomes with Microbiome Samples Expert Review (IMG/M-ER)[10]. Assignment of phylogenetic lineages was initially attempted in IMG/MER based on USEARCH similarity against all public reference genomes in IMG and the KEGG database. However, some scaffolds could not be assigned to bins while others were classified into bins not matching taxa known to be prevalent amongst the *Cephalotes* gut microbiota. To obtain more accurate information of phylogenetic binning, all scaffolds with length longer than 1000 bp from 18 metagenomes were compared to eight reference genomes of isolated gut bacteria from *C. varians* (GOLD Analysis Project ID: Ga0064586, Ga0064593, Ga0064594, Ga0064595, Ga0064585, Ga0064596, Ga0105007) and a Rhizobiales bacterium genome (accession number CP015625) using BLASTX with an e-value of 10^{-15}, identity of 70% and maxhits of 1. A scaffold was assigned to a bacterial bin if over 50% of all best BLASTX hits belonged to a single reference bacterial genome and at least 50% of the scaffold sequence was covered by the aforementioned BLASTX hits. If phylogenetic assignment by IMG/MER of a certain scaffold did not match reference genome based results, this specific scaffold was assigned to the phylogenetic group of the appropriate cultured isolate as ascertained through this BLASTx approach. Annotation of gene content was also performed by IMG/M-ER. N-metabolic pathways of gut microbiota (**Fig. 6; Supplementary Figs. 1 & 7; Supplementary Fig. 12-13**) were built manually, using KEGG and Metacyc[11] as guides

(**Supplementary Data 1, 2, 4**). All genes involved in the N-metabolic pathways of *C. varians* gut microbiota were added into a functional cart in IMG, and the "Profile & Alignment" tool in the IMG function cart was used to search those genes in non-*C. varians*-derived metagenomes. We present the nitrogen recycling and nitrogen provisioning gene presence/absence data in different bacterial taxa along with the *Cephalotes* host phylogeny[12] in **Supplementary Figure 8**. Gray bars were used in this figure to obscure cells likely affected by insufficient coverage for that taxon in the given metagenome (i.e. when total scaffold length within one metagenomic less than 50% of the total length for this taxon in the draft genome assembled for *C. varians* PL010—see below).

Sequence fragments of 16S rRNA genes with length longer than 200 bp were extracted from all 18 metagenomic libraries and 14 cultured isolate genomes. Closest relatives of each 16S rRNA sequence were identified in BLASTn searches and the top one to three BLAST hits were taken for each sequence. If the top hit was from a non-ant source, this sequence alone was selected. Up to two ant-associated sequences were selected, and the top non-ant BLAST hit was always selected. Beyond the 16S rRNA sequences from metagenomes and cultured isolates, and those from BLASTn hits, we also selected one to five sequences with close relatedness to each of the major *Cephalotes*-specific clades (based on phylogenetic placement in prior studies[13]), along with two Mollicutes sequences used as outgroups. Finally, we included a partial 16S rRNA sequence from an allantoin-dependent, urea-producing Burkholderiales derived from the sister ant genus of *Cephalotes*, *Procryptocerus*. Sequences were checked for chimeras though DECIPHER[14] and chimera filtered sequences were uploaded to the Ribosomal Database Project website for sequence alignment[15]. The alignment was then uploaded to the CIPRES web portal for maximum likelihood phylogenetic analysis using the RAxML-HPC2 on XSEDE (version 8.2.4)[16].

Amino acid sequence fragments encoded by *ureC, uraH*, and *puuD* from N-recycling pathways were extracted from each metagenomic and genomic dataset. Related homologs were identified in BLASTp searches and the top one to two for each sequence was selected. Sequences were aligned by ClustalW[17]. The alignment was uploaded to the CIPRES web portal for maximum likelihood phylogenetic analysis with bootstrapping using the RAxML-HPC BlackBox[16].

Genome binning using Anvi'o in conjunction with the CONCOCT

We used the Anvi'o metagenome visualization and annotation pipeline (version 1.2.3)[18] in conjunction with the CONCOCT differential coverage-based binning program[19] to bin assembled contigs into putative microbial genomes. These putative genomes were then manually refined to maximize completeness and minimize redundancy according to panels of single-copy marker genes as reported by Anvi'o. Briefly, reads from each of the four pooled *Cephalotes varians* metagenomic libraries were mapped against the assembled contigs using Bowtie2[20]. These read profiles were then loaded into an Anvi'o database and used for differential coverage binning with CONCOCT. All steps in this process (with the exception of manual bin refinement) were automated using the Snakemake workflow management software[21]; pipeline rules and configuration information sufficient to reproduce this analysis are made available upon request.

Amino acid sequence fragments encoded by seven protein-coding genes (*rplB, rplA, rplC, rpsB, rpsC, rpsE and tsf*) were extracted from each isolate genomic and draft genomic dataset. The concatenated alignment was uploaded to the CIPRES web portal for maximum likelihood phylogenetic analysis with bootstrapping using the RAxML-HPC BlackBox[16].

Visualization of taxonomic composition of metagenomes based on coverage and %GC

Quality and adapter-trimmed reads were mapped back to metagenome scaffolds using BWA[22] 0.7.1222 with default parameters. A Perl script sam_len_cov_gc_insert.pl (https://github.com/sujaikumar/assemblage) was used to estimate length, %GC content and average depth for each scaffold from the samfile generated by BWA. This GC-coverage file was combined with a customized file containing the information of taxonomic assignment for each scaffold using a python script make_blobology_file.py (http://static.xbase.ac.uk/files/results/nick/make_blobology_file.py). Taxon-annotated GC-coverage (TAGC) plots were then generated using scaffolds using a customized python script to visualize the contributions of different bacterial bins to the metagenome assemblies.

Fluorescence in situ hybridization

We investigated the localization of bacteria within the digestive tract of ants using fluorescence microscopy. Guts dissected from workers of *Cephalotes* sp. JGS2370 were fixed in 4% formaldehyde in PBS buffer for 2h at room temperature, then dehydrated using an ethanol gradient, and stored in 95% ethanol. After rehydration using PBS buffer with 0.03% TritonX-100, they were washed three times for 10 minutes with hybridization solution containing 30% formamide, 0.01% SDS, 0.9 M NaCl and 0.02 M Tris-HCl (pH 8.0). Hybridization was performed overnight at 37°C in hybridization solution with the addition of the universal eubacterial probe EUB338 (5'-GCTGCCTCCCGTAGGAGT-3') labeled with Cy3 at 100 nM, as well as DAPI as a counterstain. After washing with PBS, the specimens were imaged using a Leica M165FC fluorescent stereo microscope. Fluorescent microphotographs taken using the blue and green excitation filters were merged with a photograph taken under the white light. The detailed protocol is provided in[23].

Stable isotope data

Data were extracted from a prior N-isotope profiling studies[24] using graphical tools, as described and summarized previously. We also used data from supplementary files of another study that profiled *Cephalotes* N-isotopes[25]. For each locale where isotope data had been generated previously, we plotted delta ^{15}N values for *Cephalotes* next to those for other Myrmicinae ants (the sub-family containing *Cephalotes*) and ants from *Camponotus* (from the subfamily Formicinae; this genus harbors N-recycling symbionts feeding somewhat low on the food chain). Also plotted, separately for each locale, were delta ^{15}N values for sympatric plants, sap-feeding herbivores, leaf-chewing herbivores, and predators.

Assays to measure urea production (via allantoin) and urea degradation (into ammonia)

Bacteria isolated from the guts of *Cephalotes* or *Procryptocerus* ants were grown in trypticase soy broth (TSB) or TSB supplemented with 250 µM allantoin (Sigma). They were prioritized for genome sequencing based on their similarity at 16S rRNA to previously sampled bacteria. A similar rationale was used to prioritize them for *in vitro* assays.

As a proxy for the uric acid degradation pathway, we measured whether selected isolates from the Burkholderiales (**Fig. 6**; **Supplementary Data 5**) could produce urea *in vitro* and whether this production was increased by allantoin (suggesting the presence of at least part of the uric acid→urea pathway). Tubes of TSB and TSB with allantoin were inoculated from a liquid culture of the chosen isolates to an initial OD(A600) of approximately 0.05. Uninoculated control TSB and TSB + allantoin tubes were also incubated along with the inoculated samples. Sample aliquots (500 µL) were collected from each tube at various time points. The bacteria in the inoculated samples were pelleted by centrifugation at 4500xg for 10 minutes, and the liquid portion of inoculated and uninoculated samples stored at -20°C until analysis.

Urea concentrations were measured using a modified Jung assay[26]. Briefly, a solution of equal parts o-phthalaldehyde and primaquine bisphosphate (Sigma) was prepared, and 200 µL of this working solution was combined with 50 µL of samples in a 96-well assay plate. Standard concentrations of urea in TSB and TSB + 250 µM allantoin were also tested. The reaction of o-phthalaldehyde and primaquine bisphosphate with urea caused a color change, which was measured at 430 nm using a BioTek® Synergy H1 spectrophotometer. The absorbance values of the uninoculated TSB or TSB + 250 µM allantoin blank was subtracted from each standard and sample, then concentration was calculated from the standard curve, with concentrations for corrected values below that of the lowest standard (0 µM) being treated as 0 µM. The concentration of the un-inoculated samples at each time was subtracted from the corresponding concentrations of inoculated to calculate the amount of urea produced by the isolate in each media type. Average urea production at each time point was calculated and normalized by subtraction of the 0 hour average. Data were analyzed with SigmaPlot software (Systat, San Jose, CA) using a two way repeated measures ANOVA and Holm-Sidak test. Comparisons were considered statistically different if $p \leq 0.05$.

Bacteria from a range of taxa spanning multiple *Cephalotes* hosts (**Fig. 6**) were used in assays measuring ammonia production from urea. We performed a qualitative method, where bacteria were inoculated into Rapid Urea Broth (BD, Sparks, MD) containing the pH indicator phenol red. Isolates were considered positive for urea degradation if the color of the media changed from red to bright purple.

For isolates used in these assays, we generated full-length 16S rRNA sequences with Sanger sequencing. Top BLAST hits and representative sequences from particular clades were

downloaded from NCBI. These represented bacteria that had been previously found through culture-independent means (i.e. *in vivo*), typically through shallow sampling of clone libraries. Their identity or near identity to our isolates from *C. varians* and *C. rohweri* indicate that our cultured isolates are abundant core microbes. Maximum likelihood phylogenies, with bootstrapping, were conducted in the software package SeaView after sequence alignment (through the Muscle algorithm) in this same program.

Supplementary References

1. Hardy R, Burns R, Holsten RD. Applications of the acetylene-ethylene assay for measurement of nitrogen fixation. *Soil Biology and Biochemistry* **5**, 47-81 (1973).

2. Bentley BL. Nitrogen-fixation in termites - fate of newly fixed nitrogen. *J Insect Physiol* **30**, 653-655 (1984).

3. Straka J, Feldhaar H. Development of a chemically defined diet for ants. *Insect Soc* **54**, 100-104 (2007).

4. Hu Y*, et al.* By their own devices: invasive Argentine ants have shifted diet without clear aid from symbiotic microbes. *Mol Ecol* **26**, 1608-1630 (2017).

5. MacKenzie SL, Tenaschuk D. Gas-liquid chromatography of N-heptafluorobutyryl isobutyl esters of amino acids. *Journal of Chromatography A* **97**, 19-24 (1974).

6. Sanders JG, Powell S, Kronauer DJC, Vasconcelos HL, Frederickson ME, Pierce NE. Stability and phylogenetic correlation in gut microbiota: lessons from ants and apes. *Mol Ecol* **23**, 1268-1283 (2014).

7. Bolger AM, Lohse M, Usadel B. Trimmomatic: a flexible trimmer for Illumina sequence data. *Bioinformatics* **30**, 2114-2120 (2014).

8. Peng Y, Leung HCM, Yiu SM, Chin FYL. IDBA-UD: a de novo assembler for single-cell and metagenomic sequencing data with highly uneven depth. *Bioinformatics* **28**, 1420-1428 (2012).

9. Gurevich A, Saveliev V, Vyahhi N, Tesler G. QUAST: quality assessment tool for genome assemblies. *Bioinformatics* **29**, 1072-1075 (2013).

10. Markowitz VM*, et al.* IMG/M 4 version of the integrated metagenome comparative analysis system. *Nucleic Acids Res* **42**, D568-D573 (2014).

11. Caspi R*, et al.* The MetaCyc database of metabolic pathways and enzymes and the BioCyc collection of pathway/genome databases. *Nucleic Acids Res* **42**, D459-D471 (2014).

12. Price SL, Powell S, Kronauer DJC, Tran LAP, Pierce NE, Wayne RK. Renewed diversification is associated with new ecological opportunity in the Neotropical turtle ants. *J Evolution Biol* **27**, 242-258 (2014).

13. Hu Y, Lukasik P, Moreau CS, Russell JA. Correlates of gut community composition across an ant species (*Cephalotes varians*) elucidate causes and consequences of symbiotic variability. *Mol Ecol* **23**, 1284-1300 (2014).

14. Wright ES, Yilmaz LS, Noguera DR. DECIPHER, a Search-Based Approach to Chimera Identification for 16S rRNA Sequences. *Appl Environ Microb* **78**, 717-725 (2012).

15. Cole JR*, et al.* The Ribosomal Database Project: improved alignments and new tools for rRNA analysis. *Nucleic Acids Res* **37**, D141-D145 (2009).

16. Stamatakis A. RAxML version 8: a tool for phylogenetic analysis and post-analysis of large phylogenies. *Bioinformatics* **30**, 1312-1313 (2014).

17. Thompson JD, Higgins DG, Gibson TJ. CLUSTAL W: improving the sensitivity of progressive multiple sequence alignment through sequence weighting, position-specific gap penalties and weight matrix choice. *Nucleic Acids Res* **22**, 4673-4680 (1994).

18. Eren AM*, et al.* Anvi'o: an advanced analysis and visualization platformfor 'omics data. *Peerj* **3**, e1319 (2015).

19. Alneberg J*, et al.* Binning metagenomic contigs by coverage and composition. *Nat Methods* **11**, 1144-1146 (2014).

20. Langmead B, Salzberg SL. Fast gapped-read alignment with Bowtie 2. *Nat Methods* **9**, 357-359 (2012).

21. Koster J, Rahmann S. Snakemake-a scalable bioinformatics workflow engine. *Bioinformatics* **28**, 2520-2522 (2012).

22. Li H, Durbin R. Fast and accurate long-read alignment with Burrows-Wheeler transform. *Bioinformatics* **26**, 589-595 (2010).

23. Lukasik P*, et al.* The structured diversity of specialized gut symbionts of the New World army ants. *Mol Ecol* **26**, 3808-3825 (2017).

24. Davidson DW, Cook SC, Snelling RR, Chua TH. Explaining the abundance of ants in lowland tropical rainforest canopies. *Science* **300**, 969-972 (2003).

25. Tillberg CV, Holway DA, LeBrun EG, Suarez AV. Trophic ecology of invasive Argentine ants in their native and introduced ranges. *P Natl Acad Sci USA* **104**, 20856-20861 (2007).

26. Zawada RJX, Kwan P, Olszewski KL, Llinas M, Huang SG. Quantitative determination of urea concentrations in cell culture medium. *Biochem Cell Biol* **87**, 541-544 (2009).
