# Supplementary material for: Herbivorous turtle ants obtain essential nutrients from a conserved nitrogen-recycling gut microbiome
Source: Nat Commun. 2018 Mar 6;9:964. doi: 10.1038/s41467-018-03357-y (PMC5840417; doi:10.1038/s41467-018-03357-y)
Supplement: Supplementary file 3 — Description of Additional Supplementary Files [file 41467_2018_3357_MOESM3_ESM.pdf]

Description of Supplementary Files

File name: Supplementary Information
Description: Supplementary Figures, Supplementary Tables, Supplementary Methods and Supplementary Reference.

File name: Supplementary Data 1
Description: Genes from N-metabolic pathways in 18 *Cephalotes* ant gut microbiota and their distribution in different bacterial bins. B, R, O, P, X, C, F, S and H refer to Burkholderiales, Rhizobiales, Opitutales, Pseudomonadales, Xanthomonadales, Campylobacterales, Flavobacteriales, Sphingobacteriales and Hymenopera bins, respectively.

File name: Supplementary Data 2
Description: The distribution of genes from N-metabolic pathways in the 14 genomes of bacteria isolated from *C. varians* and *C. rohweri*.

File name: Supplementary Data 3
Description: Summary of scaffolds assigned to 11 bins in PL010 *C. varians* metagenome

File name: Supplementary Data 4
Description: The distribution of genes from N-metabolic pathways in the 11 bins generated based on the metagenome of *C. varians* colony PL010.

File name: Supplementary Data 5
Description: Results of *in vitro* urea production assays. Error bars indicate standard deviation.

File name: Supplementary Data 6
Description: Information of samples and fraction of the first isotopic peak abundance (M+1 abundance (fraction %)) of amino acids in the feeding experiments with ^{15}N-labelled urea and ^{13}C/^{15}N-labeled glutamate. The first isotopic peak represents the abundance of naturally occurring amino acids containing heavy isotopes.

File name: Supplementary Data 7
Description: OTU table from *C. varians* gut community samples used in feeding experiments with ^{15}N-labeled glutamate. The columns correspond to samples and rows correspond to OTUs. Numbers represent read abundance for each OTU within each library. Also indicated are taxonomic classification for each OTU.
